# Deep sequencing of gastric carcinoma reveals somatic mutations relevant to personalized medicine

**DOI:** 10.1186/1479-5876-9-119

**Published:** 2011-07-25

**Authors:** Joanna D Holbrook, Joel S Parker, Kathleen T Gallagher, Wendy S Halsey, Ashley M Hughes, Victor J Weigman, Peter F Lebowitz, Rakesh Kumar

**Affiliations:** 1 Cancer Research, Oncology R&D, Glaxosmithkline R&D, 1250 Collegeville Road, Collegeville, USA; 2 Growth, Development and Metabolism Programme, Singapore Institute of Clinical Sciences (SICS), Agency for Science Technology and Research (A*STAR), Brenner Centre for Molecular Medicine, National University of Singapore, 30 Medical Drive, 117609, Singapore; 3 Expression Analysis Inc., 4324 South Alston Avenue, Durham NC27713, USA; 4 MDR, Glaxosmithkline R&D, 1250 Collegeville Road, Collegeville, USA

## Abstract

**Background:**

Globally, gastric cancer is the second most common cause of cancer-related death, with the majority of the health burden borne by economically less-developed countries.

**Methods:**

Here, we report a genetic characterization of 50 gastric adenocarcinoma samples, using affymetrix SNP arrays and Illumina mRNA expression arrays as well as Illumina sequencing of the coding regions of 384 genes belonging to various pathways known to be altered in other cancers.

**Results:**

Genetic alterations were observed in the WNT, Hedgehog, cell cycle, DNA damage and epithelial-to-mesenchymal-transition pathways.

**Conclusions:**

The data suggests targeted therapies approved or in clinical development for gastric carcinoma would be of benefit to ~22% of the patients studied. In addition, the novel mutations detected here, are likely to influence clinical response and suggest new targets for drug discovery.

## Background

Despite recent decline of mortality rates from gastric cancer in North America and in most of Northern and Western Europe, stomach cancer remains one of the major causes of death worldwide and is common in Japan, Korea, Chile, Costa Rica, Russian Federation and other countries of the former soviet union [1]. Despite improvements in treatment modalities and screening, the prognosis of patients with gastric adenocarcinoma remains poor [2]. To understand the pathogenesis and to develop new therapeutic strategies, it is essential to dissect the molecular mechanisms that regulate the progression of gastric cancer. In particular, the oncogenic mechanisms which can be targeted by personalized medicine.

The term "oncogene addiction" to describe cancer cells highly dependent on a given oncogene or oncogenic pathway was introduced by Weinstein [3,4]. The concept underscores the development of targeted therapies which attempt to inactivate an oncogene, critical to survival of cancer cells whilst sparing normal cells which are not similarly addicted.

Several oncogenes activated at high frequency in other cancers have also been shown to be mutated in gastric cancer. It follows that marketed therapeutics targeting these oncogenes would effectively treat a proportion of gastric carcinomas, either as single agents or in combination. In January 2010, trastuzumab was approved in combination with chemotherapy for the first-line treatment of *ERBB2*-positive advanced and metastatic gastric cancer. Trastuzumab is the first targeted agent to be approved for the treatment of gastric carcinoma and an increase of 12.8% in response rate was seen with addition of Trastuzumab to chemotherapy in *ERBB2 *positive gastric adenocarcinoma [5,6]. It has been estimated that 2-27% of gastric cancers harbour *ERBB2 *amplifications and may be treated with ERBB2 inhibitors [7,8]. Similarly, overexpression of another receptor tyrosine kinase (RTK) *EGFR*, has been noted in gastric cancer and multiple trials of *EGFR *inhibitors in this cancer type are ongoing (reviewed in [9,10]). Furthermore some gastric cancers harbour DNA amplification or overexpression of the RTK *MET *[11,12] and its paralogue *MST1R *[13] and may be treated with *MET *or *MST1R *inhibitors [14-20]. Finally, *FGFR2 *over expression and amplification has been observed in a small proportion of gastric cancers (scirrhous) [21] and inhibitors have shown some efficacy in clinic [22].

Downstream of the RTKs, *KRAS *wildtype amplification and mutation has also been found in about 9-15% of gastric cancers [23,24] and may be effectively treated with MEK inhibitors [25,26]. Activation of the Pi3K/AKT/mTOR pathway has also been seen in 4-16% of gastric cancer [27-30] and so may be sensitive to PI3K inhibitors [31-34]. Similarly, cell cycle kinase *AURKA *has been shown to be activated in gastric cancer [35,36] and AURKA inhibitors in clinical development [37] may have clinical benefit.

Reports of the frequency of different types of oncogenic activation and their co-occurrence are limited. In contrast to gastrointestinonal stromal tumours (GIST) which are characterized by a high frequency of *KIT *and *PDGFRA *activation [38] and hence effectively treated in the majority by imitanib and sunitinib [39,40], gastric adenocarcinoma appears to be a molecularly heterogeneous disease with no high-frequency oncogenic perturbation discovered thus far. This is illustrated by a recent survey of somatic mutation in kinase coding genes across 14 gastric cancer cell lines and three gastric cancer tissues which discovered more than 300 novel kinase single nucleotide variations and kinase-related structural variants. However, no very frequently recurrent mutation or mutated kinase was uncovered [41].

With the aim of elucidating the potential for treatment of gastric carcinoma with targeted therapies either on the market, in development or to be discovered, we have characterized clinical gastric carcinoma samples to detect oncogene activation.

We took a global approach by assaying the samples on affymetrix SNP arrays and Illumina mRNA expression arrays. These technologies are well validated for detection of genotype, DNA copy number variation and mRNA expression profile. They are amenable to heterogeneous clinical samples. The samples were also interrogated by second generation (Illumina) sequencing. Relatively novel second generation sequencing technologies offer both increased throughput and deep sequencing capacity. The latter is especially important for characterizing cancer samples which tend to include a mixture of cell types including infiltrating normal cells, vasculature and tumour cell of different genotypes. In this study we utilized target enrichment and Illumina sequencing technology to sequence the coding regions of 384 genes. We decided to favour depth of coverage over wider coverage in order to capture mutations present in subpopulations within the tumours. Recent studies have shown cancers tend to harbour many mutations in a smaller number of signalling pathways [42,43] therefore we concentrated on genes in these pathways. We also included genes coding for proteins previously shown to affect response to targeted therapies and more likely to be successfully targeted by small molecule intervention, as our aim is to find more effective and novel ways of treating gastric carcinoma.

## Methods

### Tissue samples

DNA and RNA samples were obtained from hospitals in Russia and Vietnam according to IRB approved Protocols and with IRB approved Consent forms for molecular and genetic analysis. The medical centres themselves also have internal ethical committees with reviewed the protocol and ICFs. The samples were sourced through Tissue Solutions Ltd http://www.tissue-solutions.com/. For sample characteristics see additional file 1 table S1

### Arrays

Genotypes and copy number profiles were generated for each samples using 1 μg of DNA run on Affymetrix SNP V6 arrays using Affymetrix protocols. Copy number variation data was analysed within the ArrayStudio software http://www.Omicsoft.com. Data was normalized using Affymetrix algorithm and segmented using CBS. A transcript profile was generated for each sample using 1 μg of total RNA run on Illumnia HG-12 RNA expression arrays following the Illumina protocols. Data was analysed within the Illumina GenomeStudio software http://www.illumina.com/software/genomestudio_software.ilmn. As a data pre-processing procedure, a probe set was only retained if it has a "present" (i.e. two standard deviations above background) call in at least one of the samples. Signal values of the remaining probe sets were transformed to 2-based logarithm scale and quantile normalization was performed. DNA copy and RNA expression levels were integrated at the gene level within the ArrayStudio software http://www.Omicsoft.com. Pathway enrichment analysis was performed within the GeneGO metacore analysis suite http://www.genego.com/. All array data from this study is available in GEO http://www.ncbi.nlm.nih.gov/geo/ under series accession number GSE29999.

### Targeted deep DNA sequencing

5 μg of DNA was PCR-enriched for the coding exons of any known transcript of 384 genes of interest (additional file 2 table S2) using the Raindance platform http://www.raindancetechnologies.com/.

The resulting target libraries were sequenced using Illumnia GAII at a read-length of 54 nt. Sequence reads were mapped to the reference genome (hg18) using the BWA program [44]. Bases outside the targeted regions were ignored when summarizing coverage statistics and variant calls. SAMtools was used to parse the alignments and make genotype calls [45], and any call that deviates from reference base was regarded as a potential variant. The SAMtools package generates consensus quality and variant quality estimates to characterize the genotype calls. Accuracy of genotype calls was estimated by concordance to genotype calls from the Affymetrix 6.0 SNP microarray. Concordance matrices of samples based on both SNP and sequence data were generated to check for sample mislabelling (additional file 3 figure S1). Concordance and quantity of genotype calls were tabulated for thresholds of consensus quality, variant quality, and depth. The final set of variant calls were identified using consensus quality greater than or equal to 50 and variant quality greater than 0. To exclusively identify somatic changes, only those mutations present in the cancer sample and not detected in any of the normal samples were retained. As an additional filter for germline variants, all variants present in dbSNP and 1000 genome polymorphism datasets were removed.

### Q-PCR

Q-PCR was performed via standard protocol using Fluidigm 48*48 dynamic array. Firstly, a validation run was conducted using pooled control RNA from three specimens. Four input RNA amounts were tested (125 ng, 250 ng, 375 ng and 500 ng). Triplicate data points were obtained for the subsequently 10-point serial dilution per each condition per assay. The best overall results were at 250 or 500 ng, which yielded efficiency values ~85%. Therefore 250 ng input amount for the experimental samples. Data was produced in triplicate and mean combined. CT values were converted to abundance using standard formula abundance = 10(40-CT/3.5). Test data was normalised to housekeepers using the analysis of covariance method whereby the two housekeepers (GAPDH and beta-actin) were used to compute a robust score and the score was used as a covariate to adjust the other genes. Data analysis was performed in the Arraystudio software.

### Sanger Sequencing

Genomic DNA PCR primers were ordered from IDT (Integrated DNA Technologies Inc, Coralville, Iowa). PCR reactions were carried out using Invitrogen Platnium polymerase (Invitrogen, Carlsbad, CA). 50 ng of genomic DNA was amplified for 35 cycles at 94°C for 30 seconds, 58°C for 30 seconds and 68°C for 45 seconds. PCR products were purified using Agencourt AmPure (Agencourt Bioscience Corporation, Beverly, MA). Direct sequencing of purified PCR products with sequencing primers were performed with AB v3.1 BigDye-terminator cycle sequencing kit (Applied Biosystems, Foster City, CA) and sequencing reactions were purified using Agencourt CleanSeq (Agencourt Bioscience Corporation, Beverly, MA). The sequencing reactions were analyzed using a Genetic Analyzer 3730XL (Applied Biosystems, Foster City, CA). All sequence results data were assembled and analyzed using Codon Code Aligner (CodonCode Corporation, Dedham, MA).

## Results

### DNA and RNA amplification patterns across samples are consistent with previous studies

**Figure 1:**
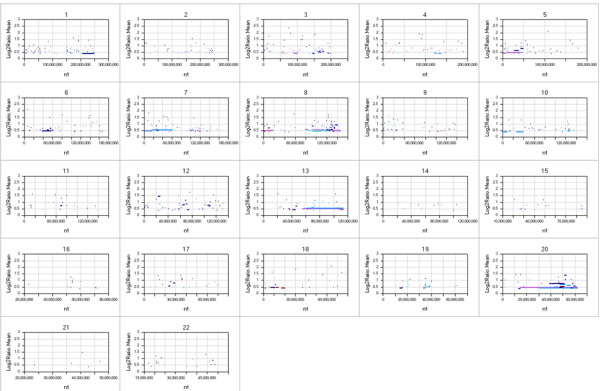
**View of CNV aberrations across all 50 gastric carcinoma samples, for each autosome**. The y-axis corresponds to the sum of the number of positive or negative changes for a particular segment with the log2 ratio of those change. Areas with increased or decreased copy number consistent throughout all the samples analysed or very large changes in few samples will show large positive and negative change sizes. Each dot or segment in figure is colored by sample. The colour code is arbitrary with each of the 50 cancer samples being assigned a colour. Amplified segments include chromosome 8q, 20q, 20p, 3q, 7p, and 1q.

Consistent with most other human cancers, copy number changes occurred across the genomes of the 50 gastric cancer samples compared to matched normal samples (Figure 1). Large regions of frequent amplification were found at chromosomal regions 8q, 13q, 20q, and 20p. Known oncogenes *MYC *and *CCNE1 *are located in the 8q and 20p amplicons, respectively and likely contribute to a growth advantage conferred by the amplification. These amplifications have been seen in prior studies in gastric cancer along with amplification of 20p for which *ZNF217 *and *TNFRSF6B *have been suggested as candidate driver genes [46].

**Figure 2:**
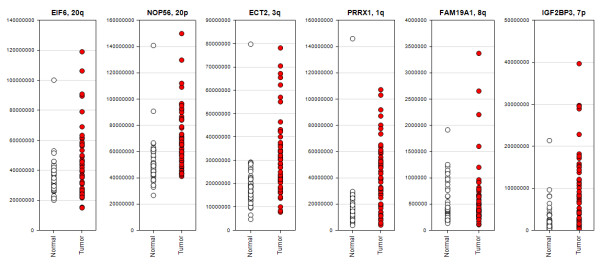
**Expression of example genes from each amplified chromosomal region across study samples confirmed by Q-PCR**. Red dots denote cancer samples and white dots denote normal samples. The y-axis denotes the mRNA abundance.

**Figure 3:**
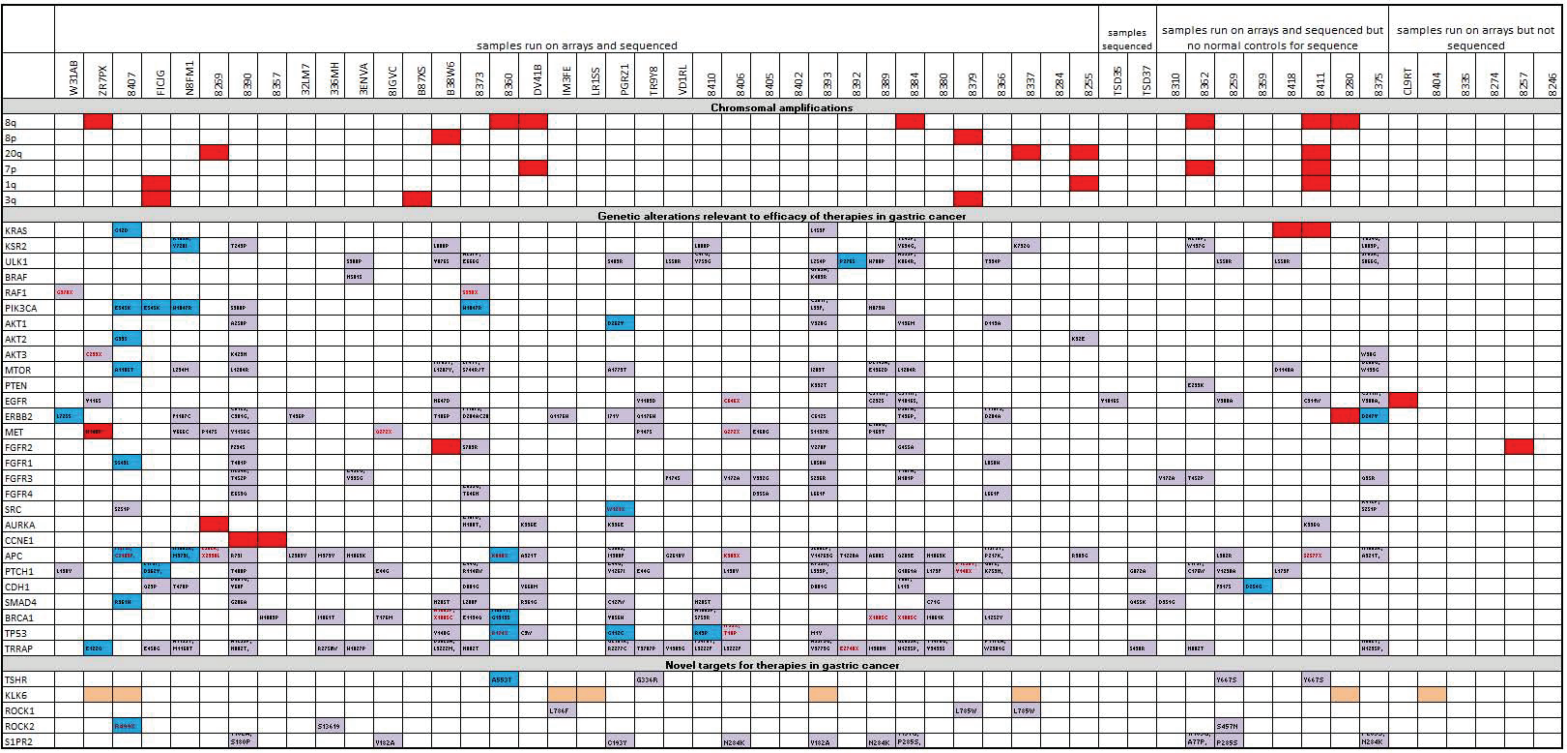
**Mutational profile of samples**. Tissue samples are displayed across the top and annotations relevant to them are in columns below. Red boxes denote DNA amplification and concordant mRNA overexpression, orange boxes denote RNA overexpression with no evidence of DNA amplification, red dots denote DNA loss. Blue boxes denote somatic nonsynonymous mutation validated by Sanger sequencing and purple boxes denote nonsynonymous somatic mutations, observed in the Illumina data with no attempt to confirm by Sanger sequencing. Amino changes are noted in the boxes and changes leading to loss or gain of a stop codon are in red text.

Concordance between DNA copy number gain and RNA expression among the cancer samples was evaluated and the top 200 genes contained within a region of frequent high DNA copy in cancer samples and which had high mRNA levels (compared to matched normal tissue) are tabulated in additional file 4 table S3. Most of the genes on this list are from chromosomal regions 20q and 8q, suggesting that these amplifications have the most effect on mRNA levels, in the minority are genes for 20p, 3q, 7p, and 1q. Figure 2 shows the RNA profiles measured by Q-PCR of an exemplar gene from each region showing general overexpression in gastric cancer, particularly in certain samples. Besides *MYC *and *CCNE1*, there are multiple genes in these regions, which could contribute to a growth advantage for the cancer cell. The biological pathways most significantly enriched for amplified and overexpressed genes are involved in regulation of translation (p = 0.000015) and DNA damage repair (p = 0.003). Samples with amplifications in these genomic regions are annotated in Figure 3. There is no discernible tendency for amplifications in these regions to co-occur or to be exclusive. In agreement with a previous study [47], the *PERLD1 *locus was amplified (within the *ERBB2 *amplicon) in sample 08280 and *MMP9 *was overexpressed but not discernibly amplified. Also in Figure 3 focal DNA amplifications with concordant RNA expression of genes likely to affect the response to targeted therapies are denoted, for example underlying data see additional file 5 figure S2.

### Sequencing data shows high concordance with genotyping

Sequencing library preparation failed for six of the original 50 cancer samples and fourteen of the original matched normal samples. Therefore two more matched pairs were added to the analysis, resulting in a dataset of 44 cancer samples, 36 with matched normal pairs (additional file 1 table S1). The targeted region included 3.28 MB across 6,547 unique exons in 384 genes (additional file 2 table S2). Median coverage of across all samples was 88.3% and dropped to 74% when requiring minimum coverage of 20. All sequencing was carried out to a minimum of 110x average read coverage across the enriched genomic regions for each sample. The reads were aligned against the human genome and variants from the reference genome were called. As a control, an analysis to compare genotyping calls from the Affymetrix V6 SNP arrays and the Illumina sequencing was performed. The regions targeted for sequencing contained 1005 loci covered by the Affymetrix V6 SNP arrays. With no filtering of the sequencing variant calls for quality metrics, the median agreement between the genotyping and sequencing results was 97.8% with a range of 65-99% (additional file 6a, Figure S3a). The raw overall genotype call concordance was 96.8%. Quality metrics were chosen to maximize the agreement between the genotyping and the sequencing calls while minimizing false negatives. The most informative metric was consensus quality and a cut-off of ≥50 resulted in loss of about 10% of the shared genotypes but an overall 2% increase in concordance to 98.7% (additional file 6b, Figure S3b). Variant genotype calls were isolated for further concordance analysis. In this set, a variant quality threshold of > 0 increased accuracy of variant genotype calls to 98.9% (additional file 6c, Figure S3c). When both quality thresholds were applied the median sample concordance is 99.5% (additional file 6d, Figure S3d) which is within the region of genotyping array error. Six samples (08362T1, 08373T2, 336MHAXA, 08337T1, 89362T2, DV41BNOH) had a concordance of < 98% and two of these (08393T2 and DV41BNOH) had a concordance of 82% and 88% respectively. Therefore with a consensus quality ≥ 50 and a variant quality > 0, the false positive rate was 0.5% and 1.6% for reference genotypes and variant genotypes, respectively (additional file 6e Figure S3e).

**Figure 4:**
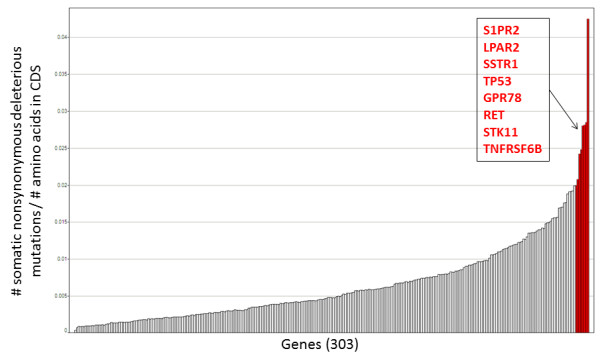
**Bar chart of rate of deleterious mutations across gene sequenced**. Genes sequenced are shown on the x-axis. The number of deleterious somatic nonsynonymous mutations observed in each gene/number of amino acids in each CDS in plotted.

From all single nucleotide changes passing the above thresholds, all variants present in any of the normal samples or in the polymorphism databases of dbSNP (v130) or 1000 genomes were assumed to be germline variants and discarded. Variants present only in the exons of cancer samples were assumed to be somatic and retained. 18,549 somatic variants were detected in total across all 44 samples (additional file 7 Table S4), 3357 were predicted to be exonic and nonsynonymous. To prioritise for mutations with functional impact we concentrate all further analyses on nonsynonymous mutations and highlighted mutations leading to loss or gain of stop codons. We have applied the SIFT algorithm [48] to predict amino acid changes that are not tolerated in evolution and so are more likely to affect the function of the protein, 1509 somatic nonsynonymous mutations have a SIFT score of < 0.05. The rate of mutations with SIFT score < 0.05 per gene, corrected for CDS length was calculated (4). Figure 4 shows, the genes with the highest concentration of low SIFT scoring mutations were *S1PR2*, *LPAR2*, *SSTR1*, *TP53*, *GPR78 *and *RET*, with S1PR2 being most extreme. There are fifteen mutations with SIFT score <0.05 across the 353aa CDS of *S1PR2*, concentrated in nine samples. *S1PR2 *also known as *EDG5 *codes for a G-protein coupled receptor of S1P and activates RhoGEF, *LARG *[49]. Little is known of its role in cancer and somatic mutations have not been observed in the 44 tissues sequenced for *S1PR2 *in the COSMIC database [50].

### Sequencing data is confirmed by Sanger sequencing

Some nonsynonymous somatic mutations were selected to be confirmed by Sanger sequencing. All mutations reported in blue in Figure 3 were confirmed by Sanger sequencing and were also confirmed to be somatic by sequencing of the wildtype sequence in the matched normal tissue (see additional file 8 Figure S4 for example sequencing traces). Although 74% were confirmed, some mutations detected in the Illumnia sequencing were not confirmed as somatic mutations by Sanger sequencing. Sixteen of the 68 (24%) mutations we attempted to confirm were present in the normal and cancer sample, these are germline mutations but not detected in any of the normal samples by Illumina sequencing and also not represented in dbSNP or 1000 genomes data. Five of the sixteen germline mutations were from cancer samples with no matched normal tissue included in the dataset, the other eleven came from cancer samples with matched normal tissue sequence included in the dataset. This evidences a rate of germline contamination not eliminated by the matched normal controls or the comparison to known polymorphism databases. It may be that the coverage of the substitutions in the normal tissue happens to be lower than in the cancer sample and so some germline mutations remain despite the somatic filters. Two of the 68 (3%) mutations we attempted to confirm were not present in the normal or cancer sample by Sanger sequencing. One cause could be false positives in the Illumnia data due to artefact; however additional file 6 Figure S3 shows the false positive rate to be low at least for those variants represented on the Affymetrix V6 arrays. Another possibility is that these are present in a subset of the sample below the sensitivity of the Sanger methodology but detected by the Illumina sequencing. Therefore, mutations reported in the Illumina sequencing are also reported in purple in Figure 3, some caution is warranted when interpreting these results as they may be germline polymorphisms or present only in a subset of the tumour sample.

### Alterations in the RAS/RAF/MEK/ERK pathway

Three tumour samples had *KRAS *genetic alterations (Figure 3) suggesting therapeutic opportunity for treatment with MEK inhibitors. One of these alterations is a G12D mutation. *KRAS *G12D mutations have been shown to initiate carcinogenesis and tumour survival [51]. Amplification and overexpression of wildtype *KRAS *was seen in the other 2 samples. *KRAS *amplification has been observed before in 5% of primary gastric cancers. Gastric cancer cell lines with wildtype *KRAS *amplification show constitutive *KRAS *activation and sensitivity to *KRAS *RNAi knockdown [24]. A novel mutation in *KRAS *was also observed; (in sample 08393) the functional consequence is unknown.

The *PIK3CA *mutation co-occurring with *KRAS *G12D, is known to affect sensitivity to MEK inhibitors [25]; in addition, novel mutations observed in this study may also have consequences for the same class of therapeutics. For instance: *KSR2 *functions as a molecular scaffold to promote ERK signalling [52,53]. Therefore, mutations in *KSR2 *such as seen in seven samples may affect sensitivity to MEK inhibitors. A second example is *ULK1*, which positively controls autophagy downstream of mTOR [54] and is mutated in fourteen samples. Autophagy is increased along with ERK phosphorylation when gastric cancer cells are treated with a proteasome inhibitor [55], therefore mutations in *ULK1 *may affect sensitivity to proteasomal inhibitor treatments such as bortezomib as a single agent or in combination with MEK inhibitors.

### Alterations in the PI3K/AKT pathway

There was substantial sequence disruption of the phosphoinositide-3-kinase (Pi3K) pathway genes in the sample set. There are a number of PI3K/AKT/mTOR inhibitors in clinical development and patients with activating mutations in the pathway are candidates for treatment [56]. *PIK3CA *mutations of known oncogenicity were found in four samples. This results in a frequency of *PIK3CA *hotspot mutation of 9%, slightly higher than previous estimates of 6% (12/185) [27] and 4.3% (4/94) [57]. The common PIK3CA hotspot mutations of known oncogenicity (E545K and H1047R) [58] were observed twice each. Another mutation in *PIK3CA *K111E, which has also been observed before in four samples in COSMIC, was observed once and potentially novel somatic mutations were observed in two more samples.

Five nonsynonymous *AKT1 *mutations were observed. Although *AKT1 *mutations are found in about 2% of all cancers, they mainly occur at amino acid 15 and the functional importance of mutation at other sites is unknown. Another nonsynonymous mutation in *AKT2 *was observed in sample 08407. *AKT2 *mutations are much rarer than *AKT1 *mutations, although an *AKT2 *mutation has been observed before in gastric carcinoma, at a 2% frequency [59]. Finally mutation of *PTEN *or *MTOR *may affect response to pathway inhibitors. Several *PTEN *mutations are noted and *MTOR *mutations are frequent.

### Alterations in Receptor Tyrosine Kinases

The receptor tyrosine kinases (RTKs) and drug targets *EGFR*, *ERBB2 *and *MET *were each amplified (log2 > 0.6) and overexpressed at the RNA level in one cancer sample. It follows that the tumours may be sensitive to the inhibitors of the amplified RTKs. In addition, multiple nonsynonymous mutations are observed in their coding regions. Downstream mutations would be expected to influence response. For instance, in the *MET *amplified sample a truncating mutation in *AKT3 *may affect sensitivity to MET inhibitors.

*FGFR2 *is amplified and RNA overexpressed in two samples, there are also multiple mutations in *FGFR1*-4. Broad range RTK inhibitors, which target FGFRs among other kinases, may be efficacious in these patients [60,61].

### Alterations in Cell Cycle Proteins

The viral oncogene homolog *SRC *is mutated in four of the tumour samples, two of the mutations are predicted to have a deleterious effect including introduction of a stop codon. This may counter-indicate SRC inhibitors. *MET *amplification is also a known resistance marker for anti-SRC therapeutics such as dasatanib [62,63]. The cell cycle related kinase, *AURKA *was amplified and overexpressed in one sample. AURKA inhibitors are in development for solid tumours [37] and may be indicated in this case. *CCNE1 *was amplified in two samples (08390 and 08357). High levels of *CCNE1 *have been shown to be frequently associated with early gastric cancer and metastasis but expression levels do not correlate with survival [64,65]. High *CCNE1 *levels have been suggested as a sensitivity marker for the gene-directed pro-drug enzyme-activated therapies [66]

### Activation of wnt pathway is common in the carcinoma samples

**Figure 5:**
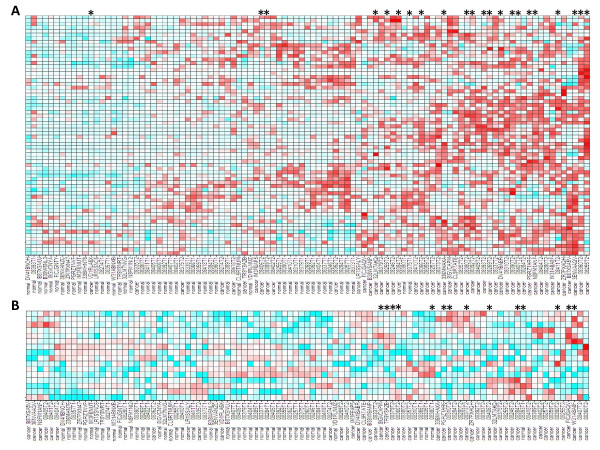
**Transcriptional signatures across samples**. Clustered heatmap showing expression of A wnt signature genes and B hedgehog signature genes, across samples in the study. All expression values are Zscore normalized. Zscore <-1 are blue, Z-score > 1 are red with a graded coloring through white at 0. Sample names are on the x-axis, they are clustered by expression pattern and samples with high signature scores are to the right. Samples with somatic nonsynonymous APC mutations (A) or PTCH1 mutations (B) and denoted by an asterisk above the heatmaps. WNT signature genes (top to bottom): *FSTL1, DACT1, CD99, LMNA, SERPINE1, TNFAIP3, GNAI2, ID2, MVP, ACTN4, CAPN1, LUZP1, MTA1, RPS19, PTPRE, AXIN2, NKD2, SFRS6, CCND1, SCAP, CPSF4, SENP2, DKK1, PRKCSH, SLC1A5, HDGF, CBX3, SCML1, PCNA, RPS11, SNRPA1, TGM2, LY6E, IFITM1, NSMAF, TCF20, BCAP31, AXIN1, AGRN, PLEKHA1, SLC2A1, CTNNB1, EIF5A, IMPDH2, GSK3B, PFN1, UBE, MAP3K11, ARHGDIA, HNRPUL1, FLOT2, GYPC, NCOA3, CENTB1, SYK, POLR2A, KRT5, DHX36, ELF1, SMG2, FGD6, MAPKAP1, LOC389435, RPL27A, SRP19, RPL39L, SFRS2IP, FUSIP1*; Hedgehog signature genes (top to bottom): *LRFN4, JAG2, RPL29, WNT5A, SNAI2, FST, MYCN, BMP4, CCND1, BMI1, CFLAR, PRDM1, GREM1, FOXF1, CCND2, CD44*.

Mutations were observed in the *APC *gene in 22 samples. APC is a tumour suppressor known to activate CTNNB1 and wnt pathway signalling, amongst other effects [67]. The wnt pathway has been previously found to be frequently activated in gastric cancer [68]. We used a transcriptional signature, generated from previous studies [69,70] and available at the Broad Institute MSigDB database to classify the study samples by their wnt transcriptional signatures. Figure 5A shows a heat map of the transcriptional levels of the WNT signature genes in the datasets. Activation of this pathway is higher in nearly all the cancer samples compared to the normal samples. Wnt inhibitors are the subject of intense investigation in pharmaceutical and academic research [71-73]. These results suggest they will have an indication in gastric cancer as well as many other cancers.

### Activation of the hedgehog pathway is also common in the carcinoma samples

*PTCH1 *is a tumour suppressor and acts as a receptor for the hedgehog ligands and inhibits the function of smoothened. When smoothened is freed, it signals intracellularly leading to the activation of the GLI transcription factors [74]. Multiple somatic mutations of *PTCH1 *are recorded in COSMIC, consistent with its tumour suppressor role. The D362Y mutation seen in this study in sample FICJG, is in the fourth transmembrane domain of PTCH1 and has been previously seen as a loss-of-function germline mutation in a patient with Gorlin syndrome, predisposing to neoplasms (numbered D513Y due to different transcript) [75]. Therefore, sample FICJG is very likely to have deregulated hedgehog signalling and does indeed have high levels of GLI target genes (as defined by [74] (Figure 5B)). Other samples also contain *PTCH1 *mutations in the Illumina sequence data, including a truncating stop codon (Y140X) in sample 08379 and have high levels of hedgehog signature genes. Hedgehog signalling has previously been shown be frequently activated in gastric cancer [76] though no genetic cause has been previously implicated. Inhibitors of the hedgehog pathway are in clinical development [77,78].

### Loss of Epithelial phenotype

Epithelial or mesenchymal status has been shown to affect response to multiple drugs [79] and samples may be more resistant due to loss of an epithelial phenotype. Both hedgehog and wnt signalling upregulate mesenchymal precursors such as *BMP4 *and mutations can lead directly to loss of epithelial phenotype. *CDH1 *is a marker of an epithelial phenotype and is often lost in gastric tumours due to the process of epithelial to mesenchymal transformation (EMT) and is a negative prognostic marker [80]. Mutations in *CDH1 *were observed in nine samples, including a D254G mutation in *CDH1 *was detected in sample 08359. A mutation at the same site (D254Y) has been recorded in COSMIC in a breast tumour and 211 somatic mutations have been observed in the 2732 samples sequenced for *CDH1 *in COSMIC. Mutation in *SMAD4 *is also likely to affect epithelial phenotype. Loss of *SMAD4 *function facilitates EMT and its re-expression reverses the process in cancer cell lines [81]. Mutations in tumour suppressor *SMAD4 *were observed in ten samples.

### Sensitivity to chemotherapy

Multiple substitutions in *BRCA1 *were observed in ten samples, including three cases of substitution of a stop codon. Germline mutations in *BRCA1 *predispose patients to breast and ovarian cancer, multiple somatic mutations have been found in tumours [82]. *BRCA1 *expression levels and polymorphic status has been shown to correlate with sensitivity to chemotherapeutics in gastric cancer [83,84]. Therefore, the observed mutations of *BRCA1 *may affect sensitivity to chemotherapy.

Another commonly mutated gene which is linked to sensitivity to chemotherapy in gastric cancer is *TP53 *[85]. Eight examples of TP53 mutation including two stop codons are seen in the dataset.

Mutations in *TRAPP *were found in 22 samples, including one mutation to a stop codon. TRRAP is a component of histone acetyltransferase complexes and is implicated in oncogenic transformation and cell fate decisions through chromatin regulation [86]. Loss of function mutations of the *Sacchromyces pombe *orthologue of *TRRAP*, cause defects in G2/M cell cycle control and resistance to *CHK1 *overexpression [87]. Mutations in *TRAPP *are likely to affect response to HDAC and CHK1 inhibitors currently approved and in trials for use as anticancer agents [88-92].

### Novel targets for therapies in gastric cancer

An additional aim of our study was to uncover novel drug targets for gastric cancer. Many novel perturbations were observed in tractable target genes, following are three examples which warrant further investigation.

Thyrotropin receptor (*TSHR*) is mutant in four samples. The A553T mutation of *TSHR *found in sample 08360, has been previously been observed in two siblings with congenital hypothyroidism and was found to be inactivating [93]. Both loss and gain of function *TSHR *mutations are often found in thyroid cancer [94]. However, a role for *TSHR *in other cancers has not been elucidated, although infrequent mutations in lung cancer are recorded in COSMIC and *TSHR *has been shown to be lost at the DNA level, in some gastric cancers [95]. Three of the four *TSHR *mutations found have very low SIFT scores and may suggest deregulation of this growth hormone pathway.

**Figure 6:**
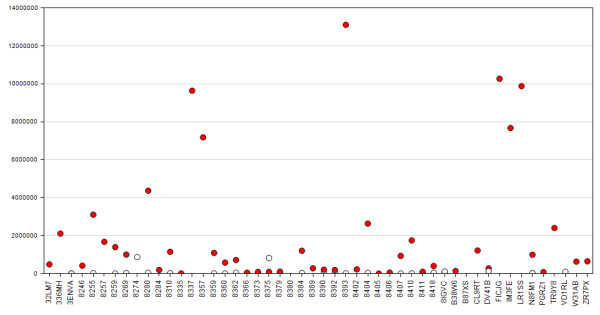
**Expression of KLK6 across study samples confirmed by q-PCR**. Red dots denote cancer samples and white dots denote normal samples. Patient IDs are arranged on the x-axis. The y-axis is the mRNA abundance.

We used the COPA algorithm [96] to identify mRNAs with outlier expression in the cancer samples. The top gene identified was *KLK6*. *KLK6 *is not detected or detected at very low levels in the normal samples, whilst its expression is very high in eleven of the cancer samples. Figure 6 shows the expression profile of *KLK6 *across the samples, confirmed by Q-PCR. *KLK6 *has previously been shown to be over expressed in gastric cancer and RNAi mediated knockdown of *KLK6 *in gastric cancer cell lines has been shown to be anti-proliferative and anti-invasive [97,98].

Finally, mutations in the Rho associated coiled-coil containing protein kinases (ROCK1 and ROCK2) are interesting in view of their role as effectors of RhoA GTPase and the recent finding that truncating mutations in *ROCK1 *(similar to the confirmed *ROCK2 *mutation in this study) are activating and lead to increased motility and adhesion in cancer cells [99].

## Discussion

Gastric adenocarcinoma rates vary widely across geographical regions, gender, ethnicity and time [100]. Diet has been shown to significantly influence gastric cancer risk as have tobacco smoking and obesity [101]. The infectious agent *Helicobacter pylori *is intimately associated with the most common types of gastric adenocarcinoma development [102]. *H. pylori *colonizes the stomach of at least half the world's population, virtually all persons infected with *H. pylori *develop gastric inflammation, which confers an increased risk for developing gastric cancer; however, only a fraction of infected individuals develop the clinical disease [103]. *H. pylori *induces generalized mutation and genomic instability in host DNA [104], which along with the complex risk profile suggests diverse routes to oncogenesis in gastric adenocarcinoma.

Therefore, an individualized personal medicine approach, measuring molecular targets in tumours and suggesting treatment regimens based on the results, is attractive. A recent study using this approach across tumour types has reported improved outcomes [105]. The trial used IHC, FISH and microarray technologies to assay levels of molecular targets in tumours, as the authors mention, second generation sequencing techniques offers a more complete picture of tumour mutagenic profile and will be even more informative in identifying sensitivity and resistance biomarkers.

## Conclusions

This study evidences previously observed perturbations of the *KRAS*, *ERBB2*, *EGFR*, *MET*, *PIK3CA, FGFR2 *and *AURKA *genes in gastric cancer and suggests some of the targeted therapies approved or in clinical development would be of benefit to 11 of the 50 patients studied. The data, also suggests that agents targeting the wnt and hedgehog pathways would be of benefit to a majority of patients. The previously undocumented DNA mutations discovered are likely to affect clinical response to marked therapeutics and may be good drug targets. Detection of these mutations was enabled by Illumina sequencing and the concordance with genotyping arrays shows its suitability for heterogeneous cancer samples. These "nextgen sequencing" techniques are just at the beginning of expanding our abilities to detect genome wide DNA mutation, DNA copy number, RNA levels and epigenetic changes, in each patient's genome. However, it remains a challenge to filter germline from somatic mutations and sort driver mutations with functional import from passenger mutations.

Whole genome studies using both Sanger and nextgen sequencing have revealed mutagenic profiles of other cancers in unprecedented completeness and detail [41,106-112]. Similar studies with large numbers of samples will be critical to fully appreciate the mutagenic diversity in gastric cancer and identify the important driver mutations. Bodies such as the ICGC (International Cancer Genomics Consortium) are currently collecting gastric adenocarcinoma samples.

Translation of these findings to clinic will require pinpointing of important mutations as well as easier access to broad diagnostic assays and clinical development of agents targeting low-frequency events [113]. Data such as that presented here, is a necessary preliminary step in delivering the maximum benefit from the major advances of targeted therapies and personalized medicine to gastric cancer patients.

## Competing interests

The authors declare that they have no competing interests.

JDH, KTG, WSH, AMH, PFL and PK are, or were employees of Glaxosmithkline plc and hold stock.

JSP and VJW are an employees of Expression Analysis Inc., who were financially compensated for some of the work in this manuscript by Glaxosmithkline.

## Authors' contributions

JDH, PFL and RK: Developed the initial idea and design of the study

JDH: managed data acquisition, analysed the array, qPCR and sequence data, interpreted the findings and drafted the manuscript.

RK: contributed to the manuscript

JSP and VJW: Analysed Illumina sequence data

KTG: Managed samples and performed translocation discovery

WSH and AMH: Carried out Sanger sequencing

All authors revised and commented on drafts of the manuscript

## Supplementary Material

Additional file 1 **Table S1: Sample characteristics**. Click here for file

Additional file 2 **Table S2: List of genes sequenced**. Click here for file

Additional file 3 **Figure S1: Concordance matrices of samples based on array and sequence data**. Click here for file

Addtional file 4 **Table S3: Top 200 genes with amplification at the DNA levels and concordant overexpression at the mRNA level**. Click here for file

Additional file 5 **Figure S2: Array data evidencing focal amplifications**. Top panels show mRNA expression data from arrays, bottom panels show log2 value for DNA abundance in genomic context as derived from SNP arrays. Click here for file

Additional file 6 **Figure S3: Comparison of genotyping calls with sequencing data**. A total of 1005 common loci were mapped between the Affymetrix 6.0 SNP microarray and the targeted regions. Concordance of genotype calls between affymetrix 6.0 SNP and SAMtools with no filters applied (top left). Application of a consensus quality filters (threshold values plotted as points) improves concordance (y-axis) but reduces the total number of calls (x-axis)(top right). A similar trend is observed for the variant quality thresholds, but at different threshold values (plotted points)(middle left). Sample concordance of genotype calls is improved with consensus quality filter >= 50 and variant quality > 0 (middle right). The total number of genotype calls stratified by reference or variant genotype, and concordance (bottom left). Click here for file

Additional file 7 **Table S4: All somatic variants detected**. Click here for file

Additional file 8 **Figure S4: Sanger sequencing traces**. Sanger sequencing traces for variants denoted by blue boxes in Figure 3 (i.e. confirmed in Illumnia and Sanger) are provided. Click here for file

## References

[B1] Bertuccio P Chatenoud L Levi F Praud D Ferlay J Negri E Malvezzi M La Vecchia C Recent patterns in gastric cancer: a global overview Int J Cancer 2009 125 666 673 10.1002/ijc.24290 19382179

[B2] Khosravi Shahi P Diaz Munoz de la Espada VM Garcia Alfonso P Encina Garcia S Izarzugaza Peron Y Arranz Cozar JL Hernandez Marin B Perez Manga G Management of gastric adenocarcinoma Clin Transl Oncol 2007 9 438 442 10.1007/s12094-007-0082-8 17652057

[B3] Weinstein IB Cancer. Addiction to oncogenes--the Achilles heal of cancer Science 2002 297 63 64 10.1126/science.1073096 12098689

[B4] Weinstein IB Joe A Oncogene addiction Cancer Res 2008 68 3077 3080 discussion 3080 10.1158/0008-5472.CAN-07-3293 18451130

[B5] Okines AF Cunningham D Trastuzumab in gastric cancer Eur J Cancer 2010 46 1949 1959 10.1016/j.ejca.2010.05.003 20542421

[B6] Bang YJ Van Cutsem E Feyereislova A Chung HC Shen L Sawaki A Lordick F Ohtsu A Omuro Y Satoh T Trastuzumab in combination with chemotherapy versus chemotherapy alone for treatment of HER2-positive advanced gastric or gastro-oesophageal junction cancer (ToGA): a phase 3, open-label, randomised controlled trial Lancet 2010 376 687 697 10.1016/S0140-6736(10)61121-X 20728210

[B7] Grabsch H Sivakumar S Gray S Gabbert HE Muller W HER2 expression in gastric cancer: Rare, heterogeneous and of no prognostic value - conclusions from 924 cases of two independent series Cell Oncol 2010 32 57 65 20208134 10.3233/CLO-2009-0497 PMC4619246

[B8] Wainberg ZA Anghel A Desai AJ Ayala R Luo T Safran B Fejzo MS Hecht JR Slamon DJ Finn RS Lapatinib, a dual EGFR and HER2 kinase inhibitor, selectively inhibits HER2-amplified human gastric cancer cells and is synergistic with trastuzumab in vitro and in vivo Clin Cancer Res 2010 16 1509 1519 10.1158/1078-0432.CCR-09-1112 20179222

[B9] Arkenau HT Gastric cancer in the era of molecularly targeted agents: current drug development strategies J Cancer Res Clin Oncol 2009 135 855 866 10.1007/s00432-009-0583-7 19363621 PMC12160194

[B10] Ku GY Ilson DH Esophagogastric cancer: targeted agents Cancer Treat Rev 2010 36 235 248 10.1016/j.ctrv.2009.12.009 20122806

[B11] Huang TJ Wang JY Lin SR Lian ST Hsieh JS Overexpression of the c-met protooncogene in human gastric carcinoma--correlation to clinical features Acta Oncol 2001 40 638 643 10.1080/028418601750444204 11669338

[B12] Kuniyasu H Yasui W Kitadai Y Yokozaki H Ito H Tahara E Frequent amplification of the c-met gene in scirrhous type stomach cancer Biochem Biophys Res Commun 1992 189 227 232 10.1016/0006-291X(92)91548-5 1333188

[B13] Zhou D Pan G Zheng C Zheng J Yian L Teng X Expression of the RON receptor tyrosine kinase and its association with gastric carcinoma versus normal gastric tissues BMC Cancer 2008 8 353 10.1186/1471-2407-8-353 19040718 PMC2629483

[B14] Buchanan SG Hendle J Lee PS Smith CR Bounaud PY Jessen KA Tang CM Huser NH Felce JD Froning KJ SGX523 is an exquisitely selective, ATP-competitive inhibitor of the MET receptor tyrosine kinase with antitumor activity in vivo Mol Cancer Ther 2009 8 3181 3190 10.1158/1535-7163.MCT-09-0477 19934279

[B15] Dai Y Siemann DW BMS-777607, a small-molecule met kinase inhibitor, suppresses hepatocyte growth factor-stimulated prostate cancer metastatic phenotype in vitro Mol Cancer Ther 2010 9 1554 1561 10.1158/1535-7163.MCT-10-0359 20515943

[B16] Munshi N Jeay S Li Y Chen CR France DS Ashwell MA Hill J Moussa MM Leggett DS Li CJ ARQ 197, a novel and selective inhibitor of the human c-Met receptor tyrosine kinase with antitumor activity Mol Cancer Ther 2010 9 1544 1553 10.1158/1535-7163.MCT-09-1173 20484018

[B17] Pan BS Chan GK Chenard M Chi A Davis LJ Deshmukh SV Gibbs JB Gil S Hang G Hatch H MK-2461, a novel multitargeted kinase inhibitor, preferentially inhibits the activated c-Met receptor Cancer Res 2010 70 1524 1533 10.1158/0008-5472.CAN-09-2541 20145145

[B18] Qian F Engst S Yamaguchi K Yu P Won KA Mock L Lou T Tan J Li C Tam D Inhibition of tumor cell growth, invasion, and metastasis by EXEL-2880 (XL880, GSK1363089), a novel inhibitor of HGF and VEGF receptor tyrosine kinases Cancer Res 2009 69 8009 8016 10.1158/0008-5472.CAN-08-4889 19808973

[B19] Smolen GA Sordella R Muir B Mohapatra G Barmettler A Archibald H Kim WJ Okimoto RA Bell DW Sgroi DC Amplification of MET may identify a subset of cancers with extreme sensitivity to the selective tyrosine kinase inhibitor PHA-665752 Proc Natl Acad Sci USA 2006 103 2316 2321 10.1073/pnas.0508776103 16461907 PMC1413705

[B20] Wang MH Padhye SS Guin S Ma Q Zhou YQ Potential therapeutics specific to c-MET/RON receptor tyrosine kinases for molecular targeting in cancer therapy Acta Pharmacol Sin 2010 31 1181 1188 10.1038/aps.2010.106 20694025 PMC4002297

[B21] Hattori Y Itoh H Uchino S Hosokawa K Ochiai A Ino Y Ishii H Sakamoto H Yamaguchi N Yanagihara K Immunohistochemical detection of K-sam protein in stomach cancer Clin Cancer Res 1996 2 1373 1381 9816310

[B22] Yashiro M Shinto O Nakamura K Tendo M Matsuoka T Matsuzaki T Kaizaki R Miwa A Hirakawa K Synergistic antitumor effects of FGFR2 inhibitor with 5-fluorouracil on scirrhous gastric carcinoma Int J Cancer 2010 126 1004 1016 19621385 10.1002/ijc.24763

[B23] Liu ZM Liu LN Li M Zhang QP Cheng SH Lu S Mutation detection of KRAS by high-resolution melting analysis in Chinese with gastric cancer Oncol Rep 2009 22 515 520 19639197 10.3892/or_00000465

[B24] Mita H Toyota M Aoki F Akashi H Maruyama R Sasaki Y Suzuki H Idogawa M Kashima L Yanagihara K A novel method, digital genome scanning detects KRAS gene amplification in gastric cancers: involvement of overexpressed wild-type KRAS in downstream signaling and cancer cell growth BMC Cancer 2009 9 198 10.1186/1471-2407-9-198 19545448 PMC2717977

[B25] Greshock J Bachman KE Degenhardt YY Jing J Wen YH Eastman S McNeil E Moy C Wegrzyn R Auger K Molecular target class is predictive of in vitro response profile Cancer Res 2010 70 3677 3686 10.1158/0008-5472.CAN-09-3788 20406975

[B26] Yoon YK Kim HP Han SW Hur HS Oh do Y Im SA Bang YJ Kim TY Combination of EGFR and MEK1/2 inhibitor shows synergistic effects by suppressing EGFR/HER3-dependent AKT activation in human gastric cancer cells Mol Cancer Ther 2009 8 2526 2536 10.1158/1535-7163.MCT-09-0300 19755509

[B27] Lee JW Soung YH Kim SY Lee HW Park WS Nam SW Kim SH Lee JY Yoo NJ Lee SH PIK3CA gene is frequently mutated in breast carcinomas and hepatocellular carcinomas Oncogene 2005 24 1477 1480 10.1038/sj.onc.1208304 15608678

[B28] Oki E Baba H Tokunaga E Nakamura T Ueda N Futatsugi M Mashino K Yamamoto M Ikebe M Kakeji Y Maehara Y Akt phosphorylation associates with LOH of PTEN and leads to chemoresistance for gastric cancer Int J Cancer 2005 117 376 380 10.1002/ijc.21170 15900596

[B29] Velho S Oliveira C Ferreira A Ferreira AC Suriano G Schwartz S Jr Duval A Carneiro F Machado JC Hamelin R Seruca R The prevalence of PIK3CA mutations in gastric and colon cancer Eur J Cancer 2005 41 1649 1654 10.1016/j.ejca.2005.04.022 15994075

[B30] Yu G Wang J Chen Y Wang X Pan J Li G Jia Z Li Q Yao JC Xie K Overexpression of phosphorylated mammalian target of rapamycin predicts lymph node metastasis and prognosis of chinese patients with gastric cancer Clin Cancer Res 2009 15 1821 1829 10.1158/1078-0432.CCR-08-2138 19223493

[B31] Cejka D Preusser M Fuereder T Sieghart W Werzowa J Strommer S Wacheck V mTOR inhibition sensitizes gastric cancer to alkylating chemotherapy in vivo Anticancer Res 2008 28 3801 3808 19189667

[B32] Cejka D Preusser M Woehrer A Sieghart W Strommer S Werzowa J Fuereder T Wacheck V Everolimus (RAD001) and anti-angiogenic cyclophosphamide show long-term control of gastric cancer growth in vivo Cancer Biol Ther 2008 7 1377 1385 10.4161/cbt.7.9.6416 18708754

[B33] Doi T Muro K Boku N Yamada Y Nishina T Takiuchi H Komatsu Y Hamamoto Y Ohno N Fujita Y Multicenter phase II study of everolimus in patients with previously treated metastatic gastric cancer J Clin Oncol 2010 28 1904 1910 10.1200/JCO.2009.26.2923 20231677

[B34] Fuereder T Jaeger-Lansky A Hoeflmayer D Preusser M Strommer S Cejka D Koehrer S Crevenna R Wacheck V mTOR inhibition by everolimus counteracts VEGF induction by sunitinib and improves anti-tumor activity against gastric cancer in vivo Cancer Lett 2010 296 249 256 10.1016/j.canlet.2010.04.015 20471160

[B35] Dar AA Zaika A Piazuelo MB Correa P Koyama T Belkhiri A Washington K Castells A Pera M El-Rifai W Frequent overexpression of Aurora Kinase A in upper gastrointestinal adenocarcinomas correlates with potent antiapoptotic functions Cancer 2008 112 1688 1698 10.1002/cncr.23371 18311783 PMC4030394

[B36] Kamada K Yamada Y Hirao T Fujimoto H Takahama Y Ueno M Takayama T Naito A Hirao S Nakajima Y Amplification/overexpression of Aurora-A in human gastric carcinoma: potential role in differentiated type gastric carcinogenesis Oncol Rep 2004 12 593 599 15289843

[B37] Traynor AM Hewitt M Liu G Flaherty KT Clark J Freedman SJ Scott BB Leighton AM Watson PA Zhao B Phase I dose escalation study of MK-0457, a novel Aurora kinase inhibitor, in adult patients with advanced solid tumors Cancer Chemother Pharmacol 2010 10.1007/s00280-010-1318-9 PMC3050703 20386909

[B38] Blanke CD Biomarkers in GIST: partly ready for prime-time use Clin Cancer Res 2009 15 5603 5605 10.1158/1078-0432.CCR-09-1563 19737947

[B39] Marrari A Trent JC George S Personalized cancer therapy for gastrointestinal stromal tumor: synergizing tumor genotyping with imatinib plasma levels Curr Opin Oncol 2010 22 336 341 10.1097/CCO.0b013e32833a6b8e 20489620

[B40] Papaetis GS Syrigos KN Targeted therapy for gastrointestinal stromal tumors: current status and future perspectives Cancer Metastasis Rev 2010 29 151 170 10.1007/s10555-010-9206-7 20112054

[B41] Zang ZJ Ong CK Cutcutache I Yu W Zhang SL Huang D Ler LD Dykema K Gan A Tao J Genetic and structural variation in the gastric cancer kinome revealed through targeted deep sequencing Cancer Res 2011 71 29 39 10.1158/0008-5472.CAN-10-1749 21097718 PMC3719377

[B42] Liu ET Functional genomics of cancer Curr Opin Genet Dev 2008 18 251 256 10.1016/j.gde.2008.07.014 18691651

[B43] Wooster R Bachman KE Catalogue, cause, complexity and cure; the many uses of cancer genome sequence Curr Opin Genet Dev 2010 20 336 341 10.1016/j.gde.2010.03.007 20382522

[B44] Li H Durbin R Fast and accurate short read alignment with Burrows-Wheeler transform Bioinformatics 2009 25 1754 1760 10.1093/bioinformatics/btp324 19451168 PMC2705234

[B45] Li H Handsaker B Wysoker A Fennell T Ruan J Homer N Marth G Abecasis G Durbin R The Sequence Alignment/Map format and SAMtools Bioinformatics 2009 25 2078 2079 10.1093/bioinformatics/btp352 19505943 PMC2723002

[B46] Buffart TE van Grieken NC Tijssen M Coffa J Ylstra B Grabsch HI van de Velde CJ Carvalho B Meijer GA High resolution analysis of DNA copy-number aberrations of chromosomes 8, 13, and 20 in gastric cancers Virchows Arch 2009 455 213 223 10.1007/s00428-009-0814-y 19697059 PMC2744787

[B47] Junnila S Kokkola A Karjalainen-Lindsberg ML Puolakkainen P Monni O Genome-wide gene copy number and expression analysis of primary gastric tumors and gastric cancer cell lines BMC Cancer 2010 10 73 10.1186/1471-2407-10-73 20187983 PMC2837868

[B48] Ng PC Henikoff S SIFT: Predicting amino acid changes that affect protein function Nucleic Acids Res 2003 31 3812 3814 10.1093/nar/gkg509 12824425 PMC168916

[B49] Medlin MD Staus DP Dubash AD Taylor JM Mack CP Sphingosine 1-phosphate receptor 2 signals through leukemia-associated RhoGEF (LARG), to promote smooth muscle cell differentiation Arterioscler Thromb Vasc Biol 2010 30 1779 1786 10.1161/ATVBAHA.110.209395 20702813 PMC2930832

[B50] Bamford S Dawson E Forbes S Clements J Pettett R Dogan A Flanagan A Teague J Futreal PA Stratton MR Wooster R The COSMIC (Catalogue of Somatic Mutations in Cancer) database and website Br J Cancer 2004 91 355 358 15188009 10.1038/sj.bjc.6601894 PMC2409828

[B51] Hingorani SR Wang L Multani AS Combs C Deramaudt TB Hruban RH Rustgi AK Chang S Tuveson DA Trp53R172H and KrasG12D cooperate to promote chromosomal instability and widely metastatic pancreatic ductal adenocarcinoma in mice Cancer Cell 2005 7 469 483 10.1016/j.ccr.2005.04.023 15894267

[B52] Costanzo-Garvey DL Pfluger PT Dougherty MK Stock JL Boehm M Chaika O Fernandez MR Fisher K Kortum RL Hong EG KSR2 is an essential regulator of AMP kinase, energy expenditure, and insulin sensitivity Cell Metab 2009 10 366 378 10.1016/j.cmet.2009.09.010 19883615 PMC2773684

[B53] Dougherty MK Ritt DA Zhou M Specht SI Monson DM Veenstra TD Morrison DK KSR2 is a calcineurin substrate that promotes ERK cascade activation in response to calcium signals Mol Cell 2009 34 652 662 10.1016/j.molcel.2009.06.001 19560418 PMC2737517

[B54] Chan EY Longatti A McKnight NC Tooze SA Kinase-inactivated ULK proteins inhibit autophagy via their conserved C-terminal domains using an Atg13-independent mechanism Mol Cell Biol 2009 29 157 171 10.1128/MCB.01082-08 18936157 PMC2612494

[B55] Wu WK Cho CH Lee CW Wu YC Yu L Li ZJ Wong CC Li HT Zhang L Ren SX Macroautophagy and ERK phosphorylation counteract the antiproliferative effect of proteasome inhibitor in gastric cancer cells Autophagy 2010 6 228 238 10.4161/auto.6.2.11042 20087064

[B56] Di Nicolantonio F Arena S Tabernero J Grosso S Molinari F Macarulla T Russo M Cancelliere C Zecchin D Mazzucchelli L Deregulation of the PI3K and KRAS signaling pathways in human cancer cells determines their response to everolimus J Clin Invest 2010 120 2858 2866 10.1172/JCI37539 20664172 PMC2912177

[B57] Li VS Wong CW Chan TL Chan AS Zhao W Chu KM So S Chen X Yuen ST Leung SY Mutations of PIK3CA in gastric adenocarcinoma BMC Cancer 2005 5 29 10.1186/1471-2407-5-29 15784156 PMC1079799

[B58] Chaussade C Cho K Mawson C Rewcastle GW Shepherd PR Functional differences between two classes of oncogenic mutation in the PIK3CA gene Biochem Biophys Res Commun 2009 381 577 581 10.1016/j.bbrc.2009.02.081 19233141

[B59] Soung YH Lee JW Nam SW Lee JY Yoo NJ Lee SH Mutational analysis of AKT1, AKT2 and AKT3 genes in common human carcinomas Oncology 2006 70 285 289 10.1159/000096289 17047397

[B60] Katoh M Genetic alterations of FGF receptors: an emerging field in clinical cancer diagnostics and therapeutics Expert Rev Anticancer Ther 2010 10 1375 1379 10.1586/era.10.128 20836672

[B61] Turner N Pearson A Sharpe R Lambros M Geyer F Lopez-Garcia MA Natrajan R Marchio C Iorns E Mackay A FGFR1 amplification drives endocrine therapy resistance and is a therapeutic target in breast cancer Cancer Res 2010 70 2085 2094 10.1158/0008-5472.CAN-09-3746 20179196 PMC2832818

[B62] Bertotti A Bracco C Girolami F Torti D Gastaldi S Galimi F Medico E Elvin P Comoglio PM Trusolino L Inhibition of Src impairs the growth of met-addicted gastric tumors Clin Cancer Res 2010 16 3933 3943 10.1158/1078-0432.CCR-10-0106 20628031

[B63] Okamoto W Okamoto I Yoshida T Okamoto K Takezawa K Hatashita E Yamada Y Kuwata K Arao T Yanagihara K Identification of c-Src as a potential therapeutic target for gastric cancer and of MET activation as a cause of resistance to c-Src inhibition Mol Cancer Ther 2010 9 1188 1197 10.1158/1535-7163.MCT-10-0002 20406949

[B64] Chang W Ma L Lin L Gu L Liu X Cai H Yu Y Tan X Zhai Y Xu X Identification of novel hub genes associated with liver metastasis of gastric cancer Int J Cancer 2009 125 2844 2853 10.1002/ijc.24699 19569046

[B65] Kouraklis G Katsoulis IE Theocharis S Tsourouflis G Xipolitas N Glinavou A Sioka C Kostakis A Does the expression of cyclin E, pRb, and p21 correlate with prognosis in gastric adenocarcinoma? Dig Dis Sci 2009 54 1015 1020 10.1007/s10620-008-0464-y 19058005

[B66] Abate-Daga D Garcia-Rodriguez L Sumoy L Fillat C Cell cycle control pathways act as conditioning factors for TK/GCV sensitivity in pancreatic cancer cells Biochim Biophys Acta 2010 1803 1175 1185 10.1016/j.bbamcr.2010.06.009 20599444

[B67] Phelps RA Broadbent TJ Stafforini DM Jones DA New perspectives on APC control of cell fate and proliferation in colorectal cancer Cell Cycle 2009 8 2549 2556 10.4161/cc.8.16.9278 19597346

[B68] Ooi CH Ivanova T Wu J Lee M Tan IB Tao J Ward L Koo JH Gopalakrishnan V Zhu Y Oncogenic pathway combinations predict clinical prognosis in gastric cancer PLoS Genet 2009 5 e1000676 10.1371/journal.pgen.1000676 19798449 PMC2748685

[B69] Bild AH Yao G Chang JT Wang Q Potti A Chasse D Joshi MB Harpole D Lancaster JM Berchuck A Oncogenic pathway signatures in human cancers as a guide to targeted therapies Nature 2006 439 353 357 10.1038/nature04296 16273092

[B70] Willert J Epping M Pollack JR Brown PO Nusse R A transcriptional response to Wnt protein in human embryonic carcinoma cells BMC Dev Biol 2002 2 8 10.1186/1471-213X-2-8 12095419 PMC117803

[B71] Chen W Chen M Barak LS Development of small molecules targeting the Wnt pathway for the treatment of colon cancer: a high-throughput screening approach Am J Physiol Gastrointest Liver Physiol 2010 299 G293 300 10.1152/ajpgi.00005.2010 20508156 PMC2928541

[B72] Ewan K Pajak B Stubbs M Todd H Barbeau O Quevedo C Botfield H Young R Ruddle R Samuel L A useful approach to identify novel small-molecule inhibitors of Wnt-dependent transcription Cancer Res 2010 70 5963 5973 10.1158/0008-5472.CAN-10-1028 20610623 PMC2912498

[B73] Huang SM Mishina YM Liu S Cheung A Stegmeier F Michaud GA Charlat O Wiellette E Zhang Y Wiessner S Tankyrase inhibition stabilizes axin and antagonizes Wnt signalling Nature 2009 461 614 620 10.1038/nature08356 19759537

[B74] Katoh Y Katoh M Hedgehog target genes: mechanisms of carcinogenesis induced by aberrant hedgehog signaling activation Curr Mol Med 2009 9 873 886 10.2174/156652409789105570 19860666

[B75] Wicking C Shanley S Smyth I Gillies S Negus K Graham S Suthers G Haites N Edwards M Wainwright B Chenevix-Trench G Most germ-line mutations in the nevoid basal cell carcinoma syndrome lead to a premature termination of the PATCHED protein, and no genotype-phenotype correlations are evident Am J Hum Genet 1997 60 21 26 8981943 PMC1712561

[B76] Ma X Chen K Huang S Zhang X Adegboyega PA Evers BM Zhang H Xie J Frequent activation of the hedgehog pathway in advanced gastric adenocarcinomas Carcinogenesis 2005 26 1698 1705 10.1093/carcin/bgi130 15905200

[B77] Dierks C GDC-0449--targeting the hedgehog signaling pathway Recent Results Cancer Res 2010 184 235 238 10.1007/978-3-642-01222-8_17 20072843

[B78] Amin SH Tibes R Kim JE Hybarger CP Hedgehog antagonist GDC-0449 is effective in the treatment of advanced basal cell carcinoma Laryngoscope 2010 10.1002/lary.21145 20927781

[B79] Sabbah M Emami S Redeuilh G Julien S Prevost G Zimber A Ouelaa R Bracke M De Wever O Gespach C Molecular signature and therapeutic perspective of the epithelial-to-mesenchymal transitions in epithelial cancers Drug Resist Updat 2008 11 123 151 10.1016/j.drup.2008.07.001 18718806

[B80] Katoh M Epithelial-mesenchymal transition in gastric cancer (Review) Int J Oncol 2005 27 1677 1683 16273224

[B81] Pohl M Radacz Y Pawlik N Schoeneck A Baldus SE Munding J Schmiegel W Schwarte-Waldhoff I Reinacher-Schick A SMAD4 mediates mesenchymal-epithelial reversion in SW480 colon carcinoma cells Anticancer Res 2010 30 2603 2613 20682989

[B82] Linger RJ Kruk PA BRCA1 16 years later: risk-associated BRCA1 mutations and their functional implications FEBS J 2010 277 3086 3096 10.1111/j.1742-4658.2010.07735.x 20608970

[B83] Shim HJ Yun JY Hwang JE Bae WK Cho SH Lee JH Kim HN Shin MH Kweon SS Kim HJ Chung IJ BRCA1 and XRCC1 polymorphisms associated with survival in advanced gastric cancer treated with taxane and cisplatin Cancer Sci 2010 101 1247 1254 10.1111/j.1349-7006.2010.01514.x 20331623 PMC11158840

[B84] Wang L Wei J Qian X Yin H Zhao Y Yu L Wang T Liu B ERCC1 and BRCA1 mRNA expression levels in metastatic malignant effusions is associated with chemosensitivity to cisplatin and/or docetaxel BMC Cancer 2008 8 97 10.1186/1471-2407-8-97 18402708 PMC2394535

[B85] Yashiro M Inoue T Nishioka N Matsuoka T Boland CR Hirakawa K Allelic imbalance at p53 and microsatellite instability are predictive markers for resistance to chemotherapy in gastric carcinoma Ann Surg Oncol 2009 16 2926 2935 10.1245/s10434-009-0590-6 19597886 PMC2865194

[B86] Murr R Vaissiere T Sawan C Shukla V Herceg Z Orchestration of chromatin-based processes: mind the TRRAP Oncogene 2007 26 5358 5372 10.1038/sj.onc.1210605 17694078

[B87] Calonge TM Eshaghi M Liu J Ronai Z O'Connell MJ Transformation/transcription domain-associated protein (TRRAP)-mediated regulation of Wee1 Genetics 2010 185 81 93 10.1534/genetics.110.114769 20194963 PMC2870978

[B88] Campas-Moya C Romidepsin for the treatment of cutaneous T-cell lymphoma Drugs Today (Barc) 2009 45 787 795 20126671 10.1358/dot.2009.45.11.1437052

[B89] Copeland A Buglio D Younes A Histone deacetylase inhibitors in lymphoma Curr Opin Oncol 22 431 436 20683267 10.1097/CCO.0b013e32833d5954

[B90] Janetka JW Ashwell S Checkpoint kinase inhibitors: a review of the patent literature Expert Opin Ther Pat 2009 19 165 197 10.1517/13543770802653622 19441917

[B91] Kavanaugh SM White LA Kolesar JM Vorinostat: A novel therapy for the treatment of cutaneous T-cell lymphoma Am J Health Syst Pharm 2010 67 793 797 10.2146/ajhp090247 20479100

[B92] Morgan MA Parsels LA Zhao L Parsels JD Davis MA Hassan MC Arumugarajah S Hylander-Gans L Morosini D Simeone DM Mechanism of radiosensitization by the Chk1/2 inhibitor AZD7762 involves abrogation of the G2 checkpoint and inhibition of homologous recombinational DNA repair Cancer Res 2010 70 4972 4981 10.1158/0008-5472.CAN-09-3573 20501833 PMC2889008

[B93] Abramowicz MJ Duprez L Parma J Vassart G Heinrichs C Familial congenital hypothyroidism due to inactivating mutation of the thyrotropin receptor causing profound hypoplasia of the thyroid gland J Clin Invest 1997 99 3018 3024 10.1172/JCI119497 9185526 PMC508154

[B94] Garcia-Jimenez C Santisteban P TSH signalling and cancer Arq Bras Endocrinol Metabol 2007 51 654 671 10.1590/S0004-27302007000500003 17891229

[B95] van Dekken H Geelen E Dinjens WN Wijnhoven BP Tilanus HW Tanke HJ Rosenberg C Comparative genomic hybridization of cancer of the gastroesophageal junction: deletion of 14Q31-32. 1 discriminates between esophageal (Barrett's) and gastric cardia adenocarcinomas Cancer Res 1999 59 748 752 9973227

[B96] Hanauer DA Rhodes DR Sinha-Kumar C Chinnaiyan AM Bioinformatics approaches in the study of cancer Curr Mol Med 2007 7 133 141 10.2174/156652407779940431 17311538

[B97] Henkhaus RS Gerner EW Ignatenko NA Kallikrein 6 is a mediator of K-RAS-dependent migration of colon carcinoma cells Biol Chem 2008 389 757 764 10.1515/BC.2008.087 18627290 PMC3574817

[B98] Nagahara H Mimori K Utsunomiya T Barnard GF Ohira M Hirakawa K Mori M Clinicopathologic and biological significance of kallikrein 6 overexpression in human gastric cancer Clin Cancer Res 2005 11 6800 6806 10.1158/1078-0432.CCR-05-0943 16203767

[B99] Lochhead PA Wickman G Mezna M Olson MF Activating ROCK1 somatic mutations in human cancer Oncogene 2010 29 2591 2598 10.1038/onc.2010.3 20140017

[B100] Volk J Parsonnet J Wang T, Fox J, Giraud A Epidemiology od Gastric Cancer and Helicobacter pylori The Biology of Gastric Cancers 2009 Springer

[B101] Compare D Rocco A Nardone G Risk factors in gastric cancer Eur Rev Med Pharmacol Sci 2010 14 302 308 20496539

[B102] Marshall BJ McGechie DB Rogers PA Glancy RJ Pyloric Campylobacter infection and gastroduodenal disease Med J Aust 1985 142 439 444 3982346 10.5694/j.1326-5377.1985.tb113444.x

[B103] Israel DA Peek RM Wang T, Fox J, Giraud A The role of Helicobacter pylori virulence factors in rodent and primate models of disease The Biology of Gastric Cancers 2009 Springer 403 423

[B104] Machado AM Figueiredo C Seruca R Rasmussen LJ Helicobacter pylori infection generates genetic instability in gastric cells Biochim Biophys Acta 2010 1806 58 65 20122996 10.1016/j.bbcan.2010.01.007

[B105] Von Hoff DD Stephenson JJ Jr Rosen P Loesch DM Borad MJ Anthony S Jameson G Brown S Cantafio N Richards DA Pilot Study Using Molecular Profiling of Patients' Tumors to Find Potential Targets and Select Treatments for Their Refractory Cancers J Clin Oncol 2010 10.1200/JCO.2009.26.5983 20921468

[B106] Comprehensive genomic characterization defines human glioblastoma genes and core pathways Nature 2008 455 1061 1068 10.1038/nature07385 18772890 PMC2671642

[B107] Jones S Zhang X Parsons DW Lin JC Leary RJ Angenendt P Mankoo P Carter H Kamiyama H Jimeno A Core signaling pathways in human pancreatic cancers revealed by global genomic analyses Science 2008 321 1801 1806 10.1126/science.1164368 18772397 PMC2848990

[B108] Mardis ER Ding L Dooling DJ Larson DE McLellan MD Chen K Koboldt DC Fulton RS Delehaunty KD McGrath SD Recurring mutations found by sequencing an acute myeloid leukemia genome N Engl J Med 2009 361 1058 1066 10.1056/NEJMoa0903840 19657110 PMC3201812

[B109] Pleasance ED Stephens PJ O'Meara S McBride DJ Meynert A Jones D Lin ML Beare D Lau KW Greenman C A small-cell lung cancer genome with complex signatures of tobacco exposure Nature 2010 463 184 190 10.1038/nature08629 20016488 PMC2880489

[B110] Shah SP Morin RD Khattra J Prentice L Pugh T Burleigh A Delaney A Gelmon K Guliany R Senz J Mutational evolution in a lobular breast tumour profiled at single nucleotide resolution Nature 2009 461 809 813 10.1038/nature08489 19812674

[B111] Weir BA Woo MS Getz G Perner S Ding L Beroukhim R Lin WM Province MA Kraja A Johnson LA Characterizing the cancer genome in lung adenocarcinoma Nature 2007 450 893 898 10.1038/nature06358 17982442 PMC2538683

[B112] Wood LD Parsons DW Jones S Lin J Sjoblom T Leary RJ Shen D Boca SM Barber T Ptak J The genomic landscapes of human breast and colorectal cancers Science 2007 318 1108 1113 10.1126/science.1145720 17932254

[B113] Simon R Clinical trial designs for evaluating the medical utility of prognostic and predictive biomarkers in oncology Per Med 2010 7 33 47 10.2217/pme.09.49 20383292 PMC2851173

