# Supplementary material for: Deep sequencing of gastric carcinoma reveals somatic mutations relevant to personalized medicine
Source: J Transl Med. 2011 Jul 25;9:119. doi: 10.1186/1479-5876-9-119 (PMC3152520; doi:10.1186/1479-5876-9-119)

Cluster analysis of samples based on Illumina sequencing data

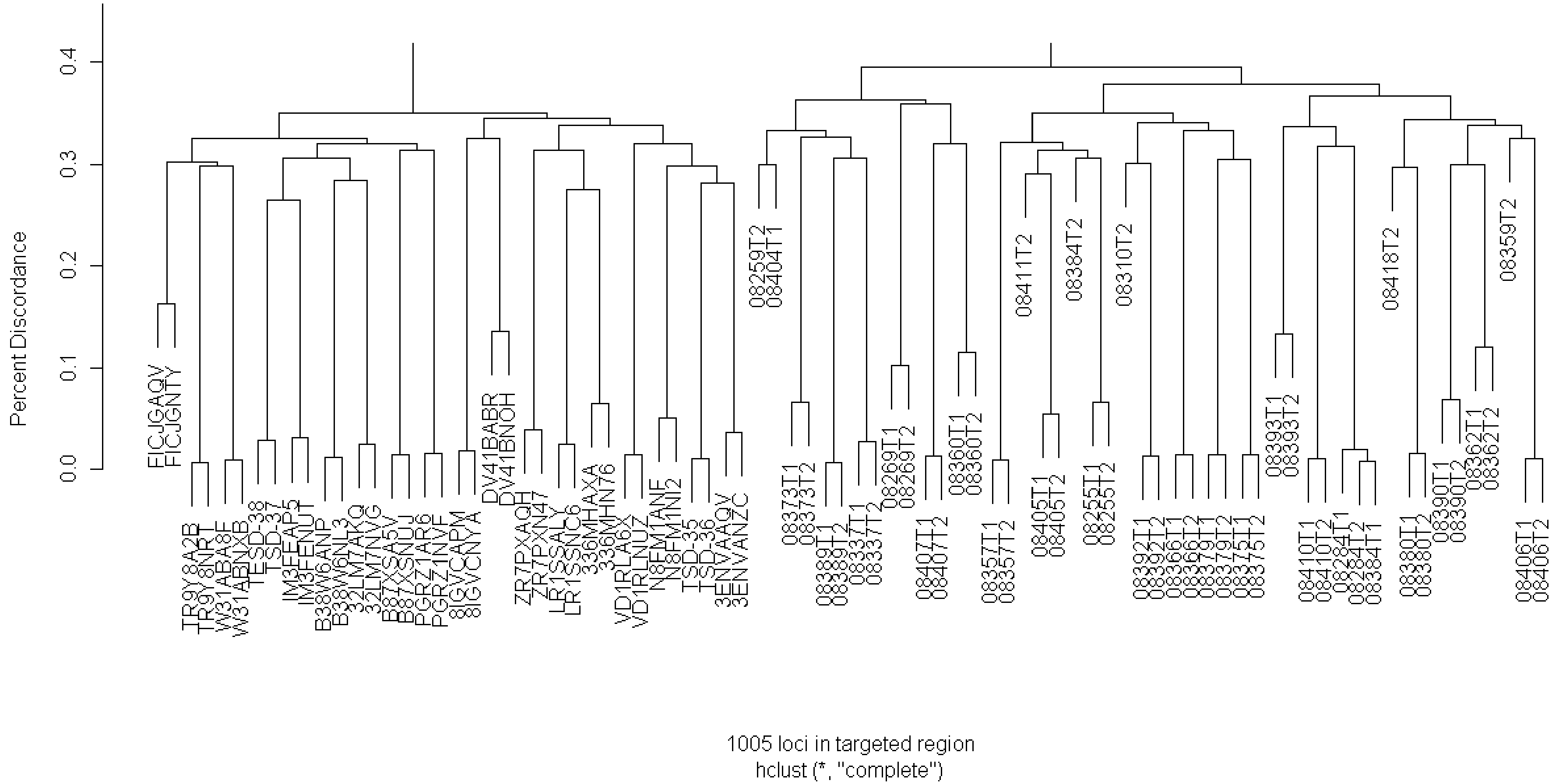

Cluster analysis of samples based on genotyping array data [1005 loci in targeted region]

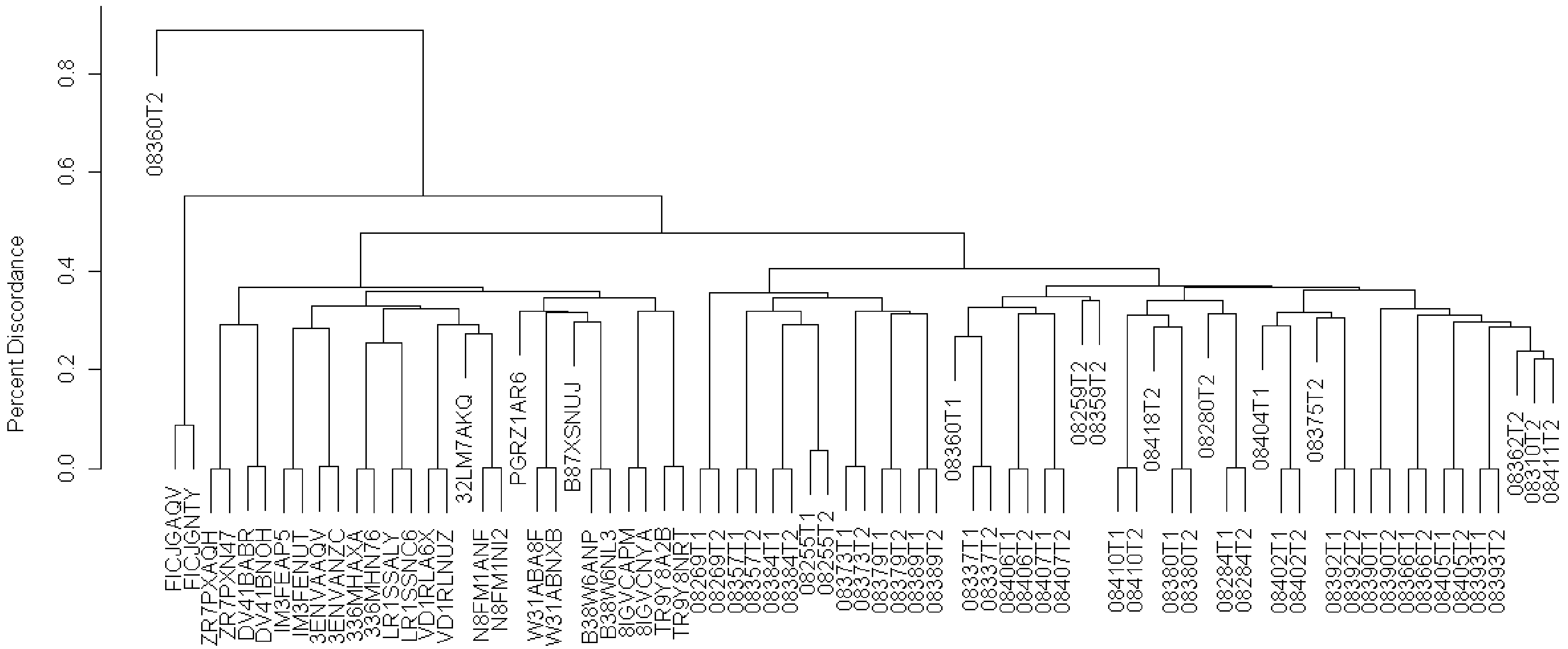

Supplement: Additional file 3 — Figure S1: Concordance matrices of samples based on array and sequence data. [file 1479-5876-9-119-S3.PDF]
