# Supplementary figures and images for: Deep sequencing of gastric carcinoma reveals somatic mutations relevant to personalized medicine
Source: J Transl Med. 2011 Jul 25;9:119. doi: 10.1186/1479-5876-9-119 (PMC3152520; doi:10.1186/1479-5876-9-119)

**Figure S1**

**EGFR**

**
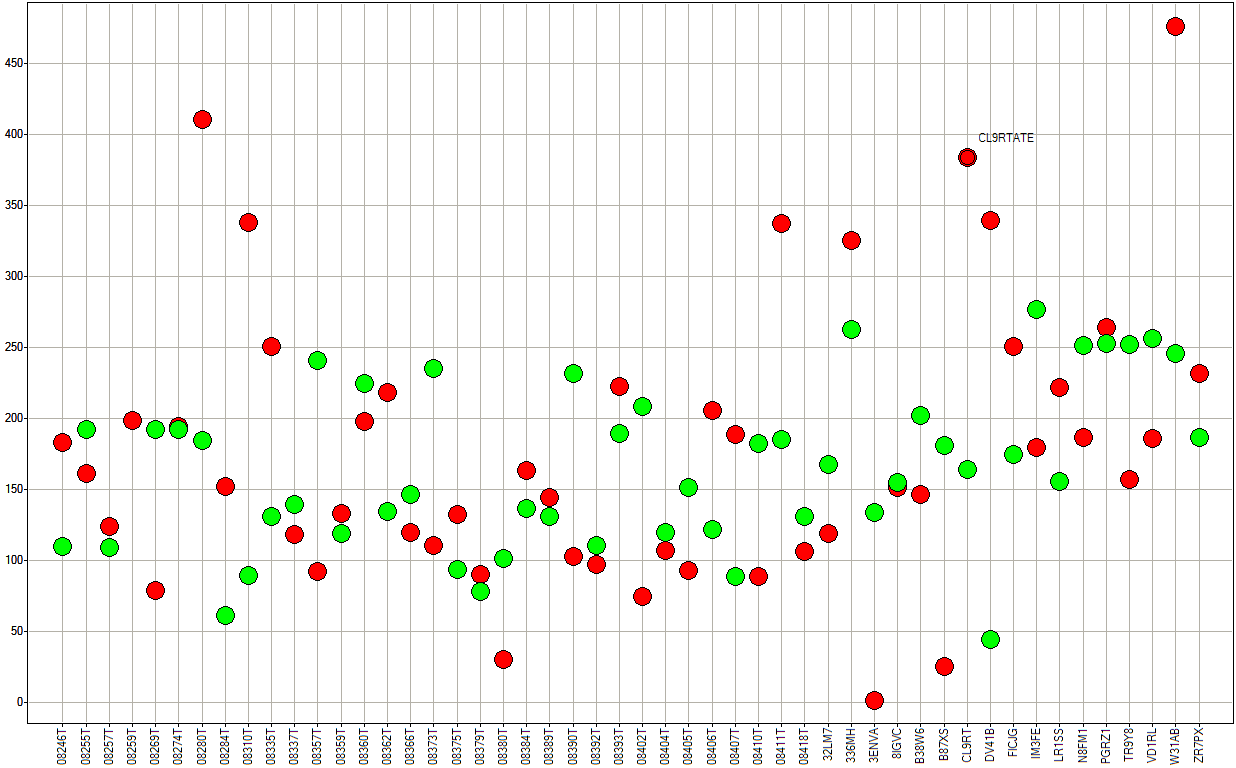
**

**
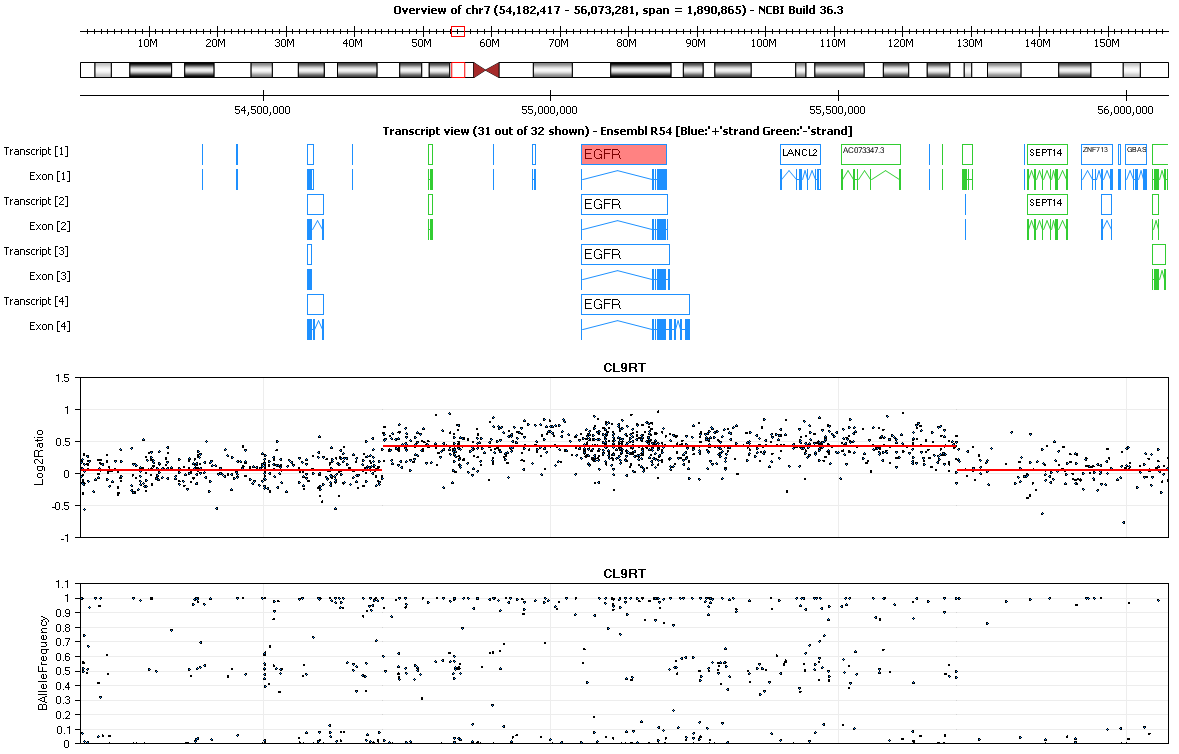
**

**ERBB2**

**
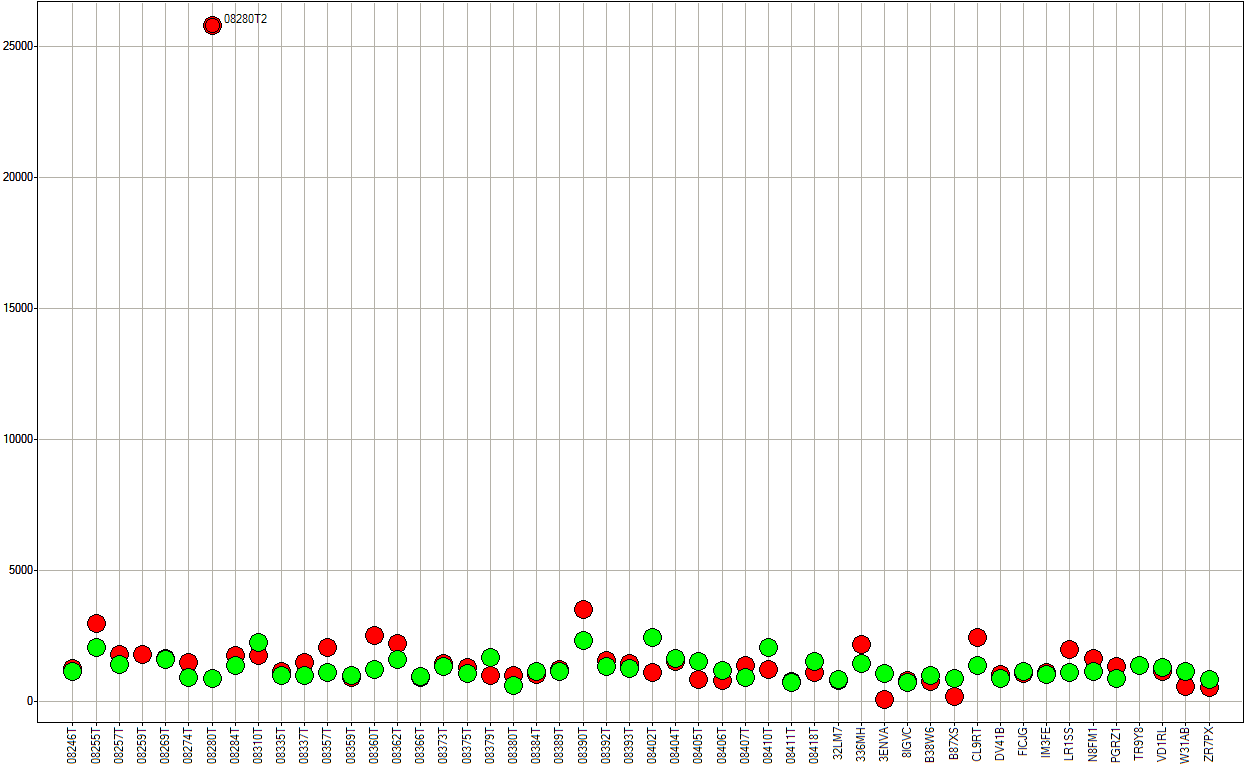
**

**
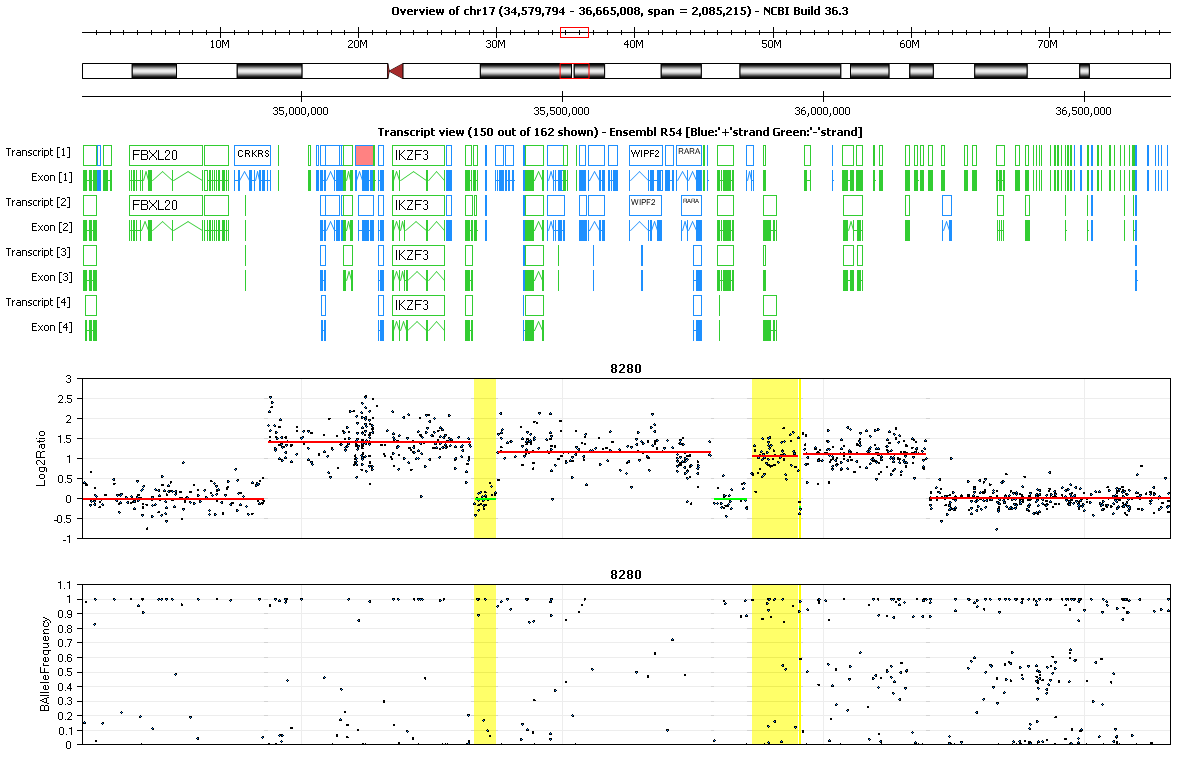
**

**MET**

**
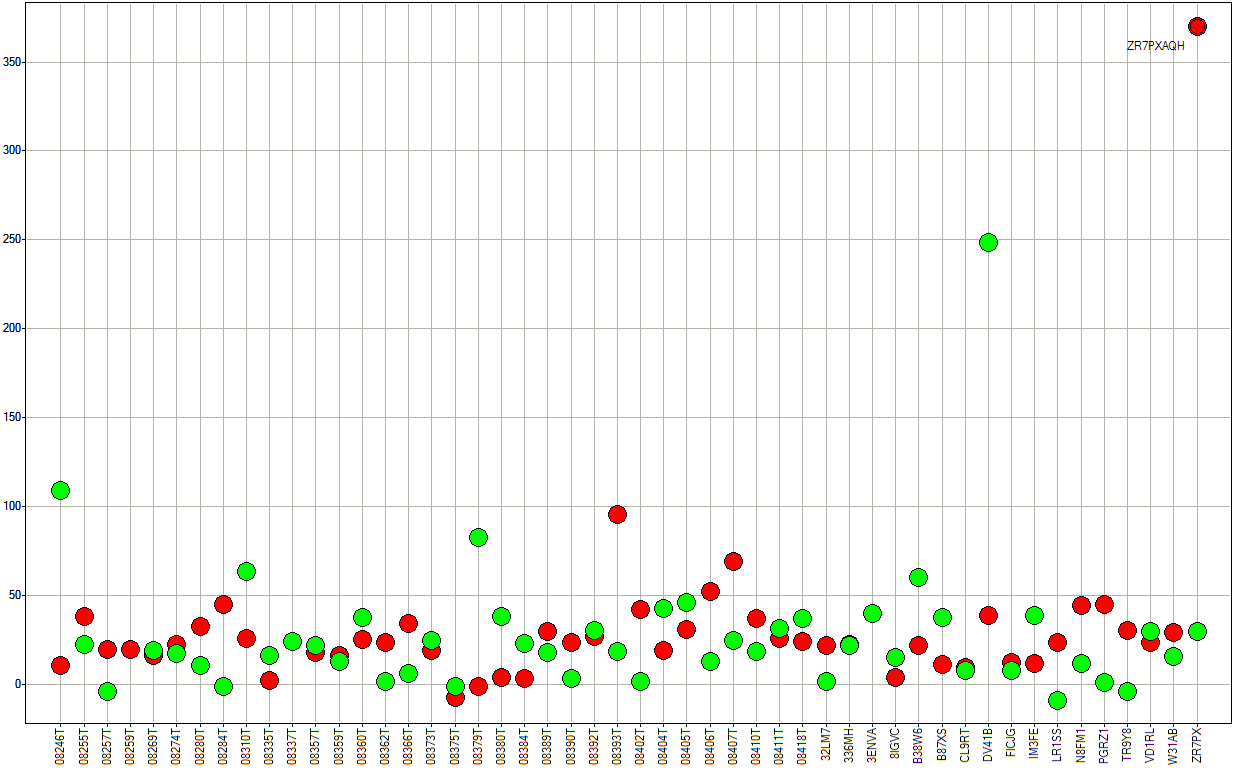
**

**
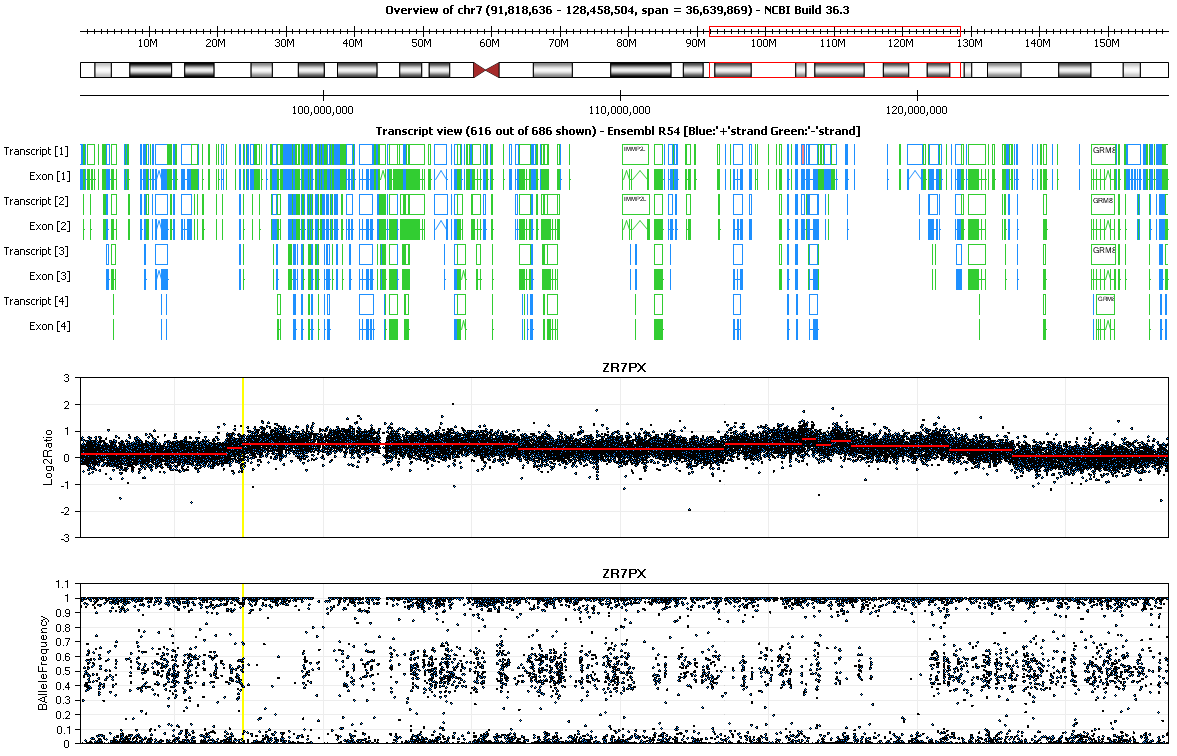
**

**KRAS**

**
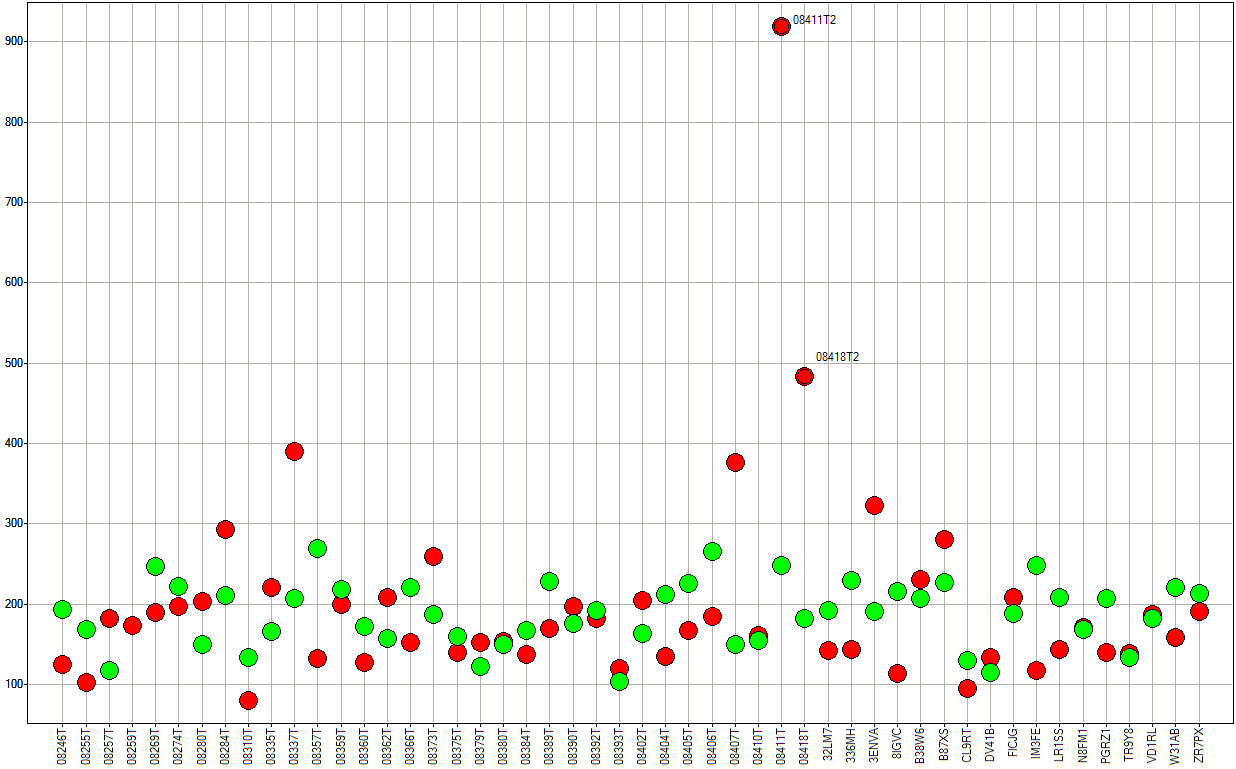
**

**
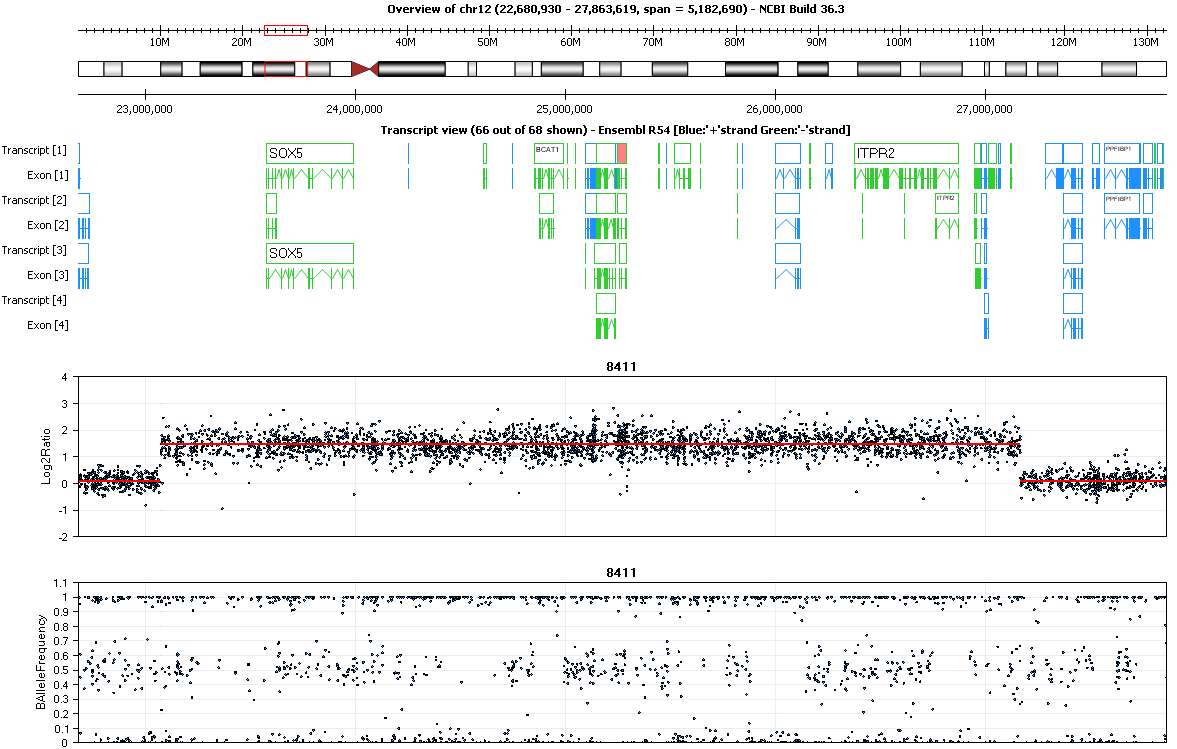
**

**
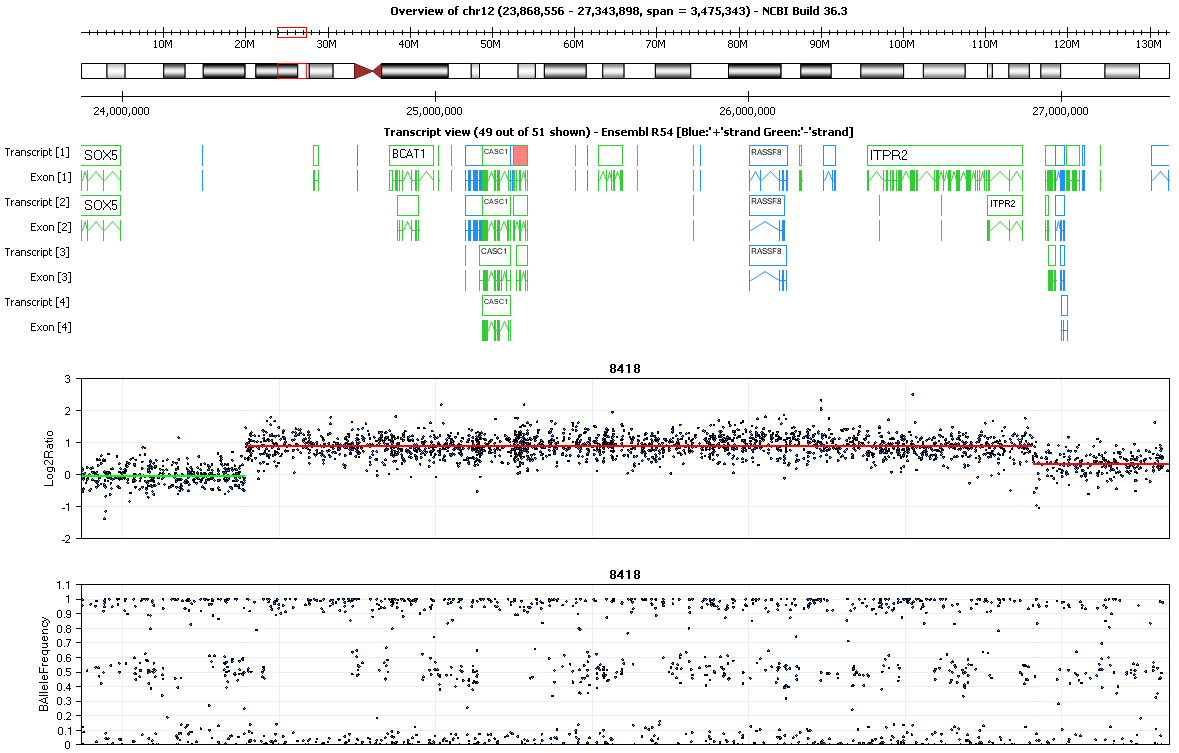
**

**AURKA**

**
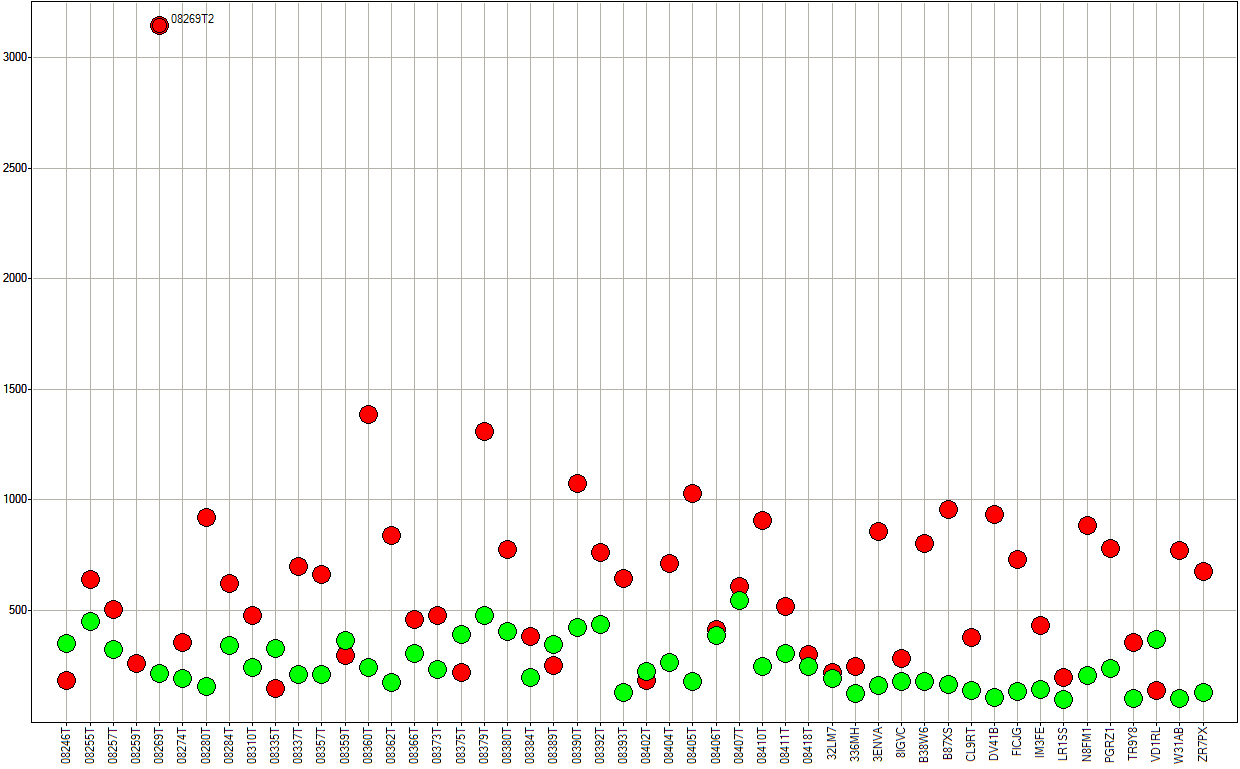
**

**
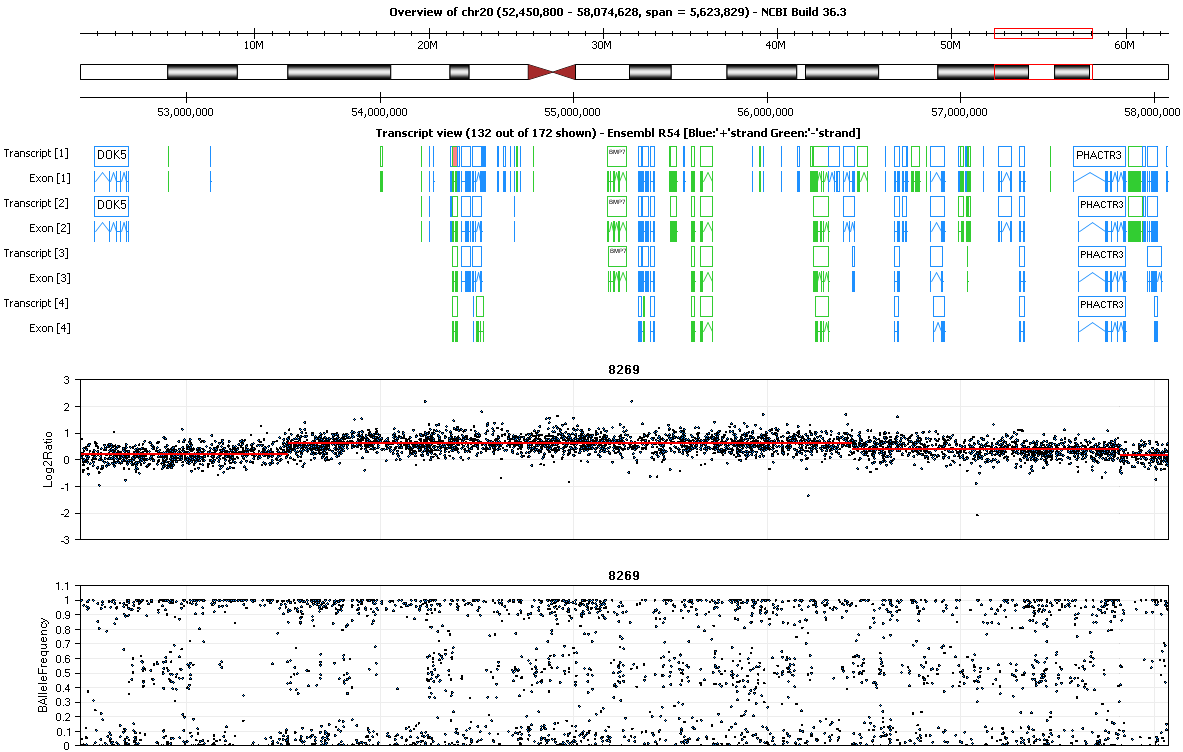
**

**CCNE1**

**
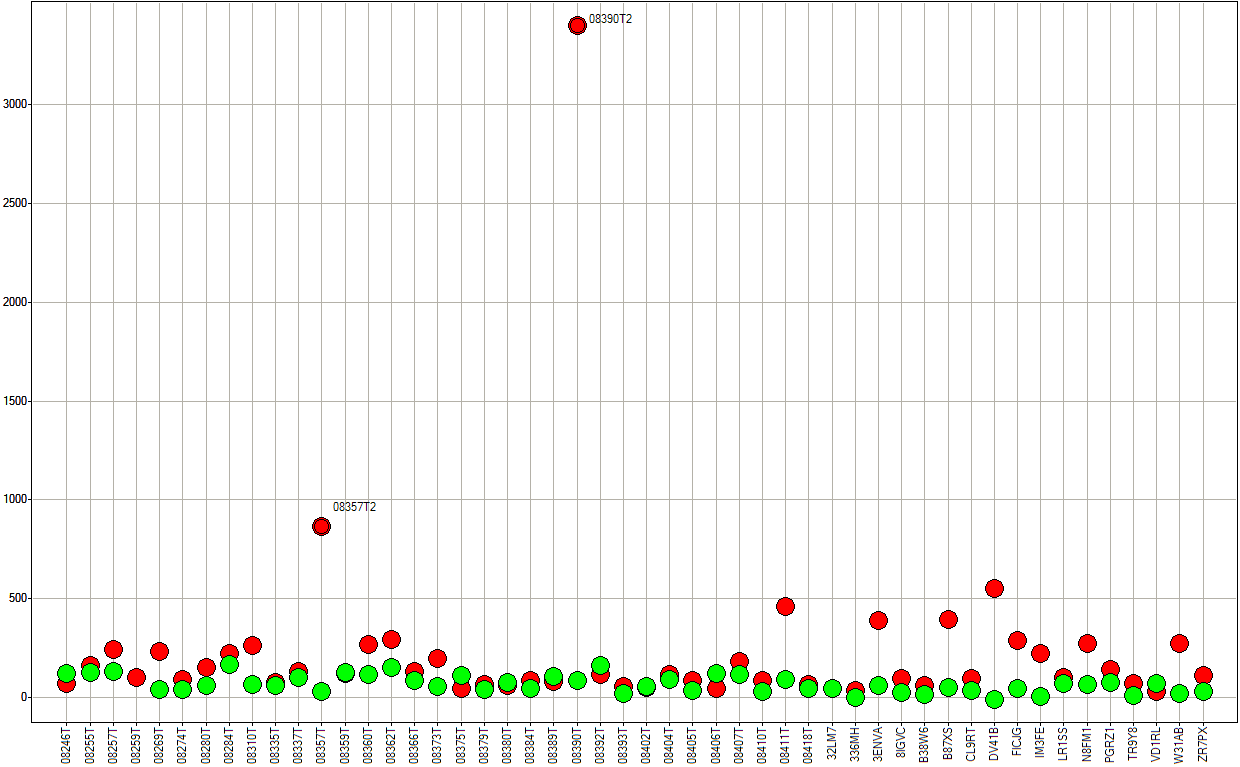
**

**
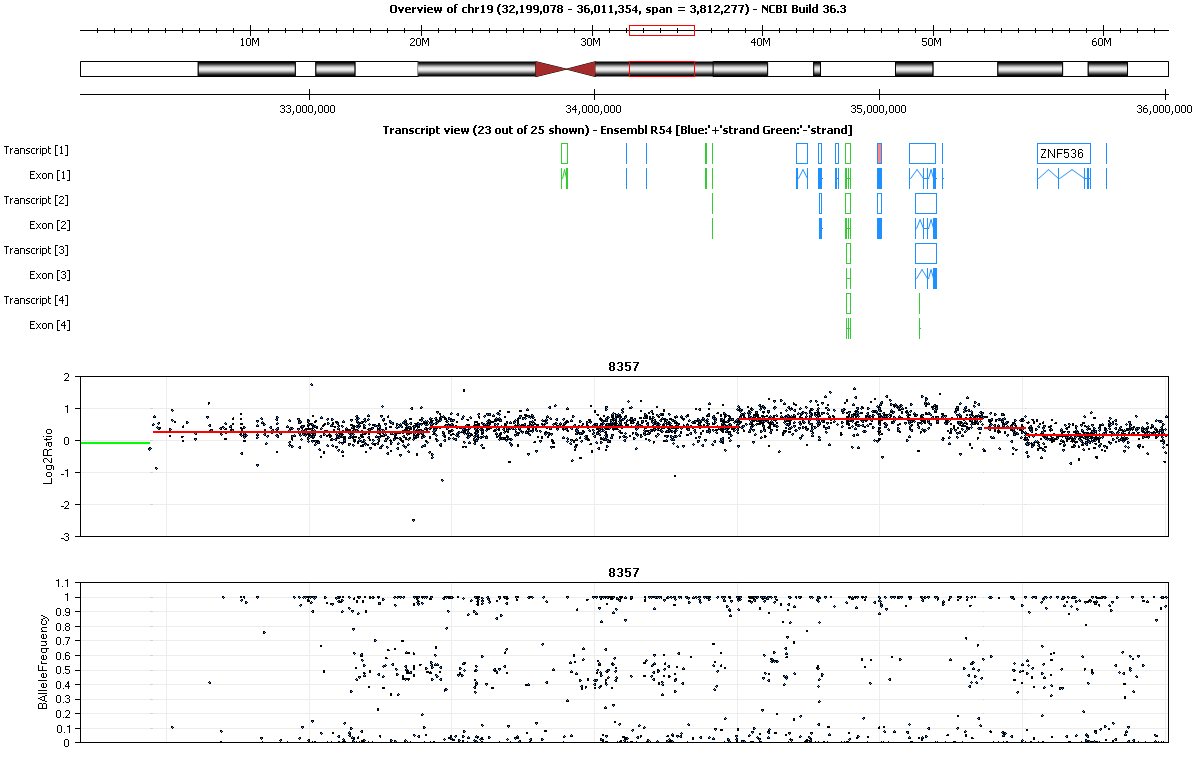
**

**
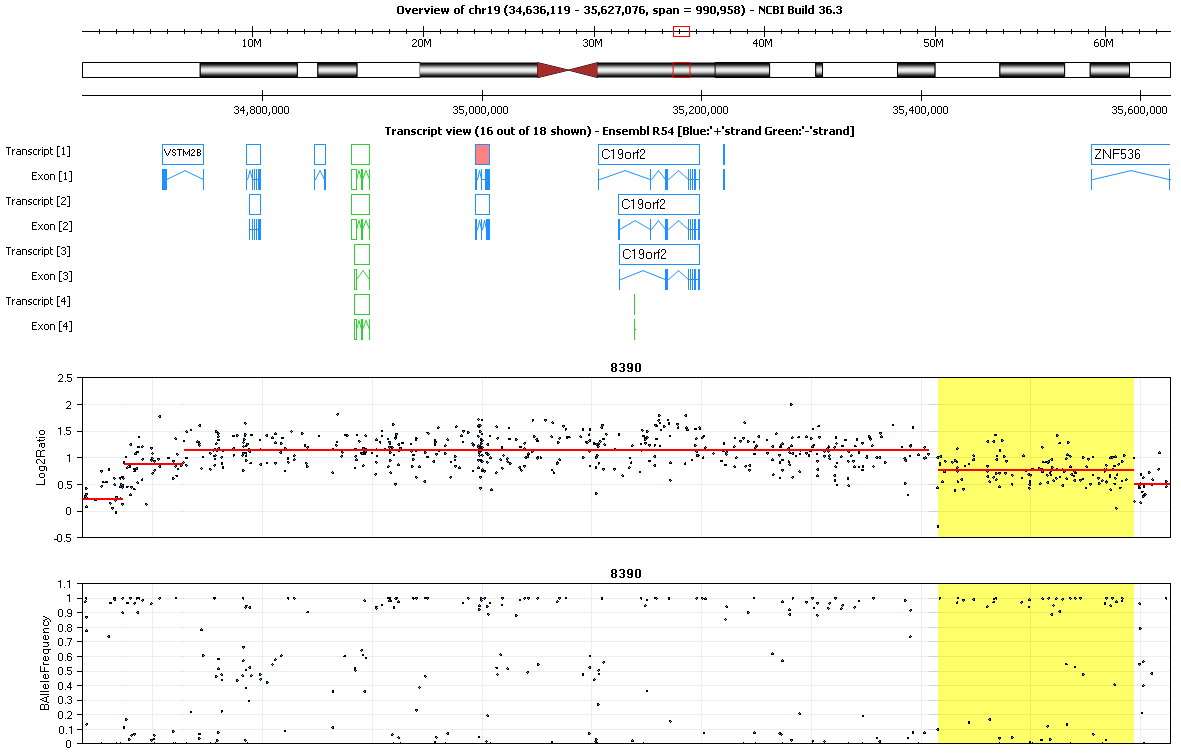
**

**PERLD1**

**
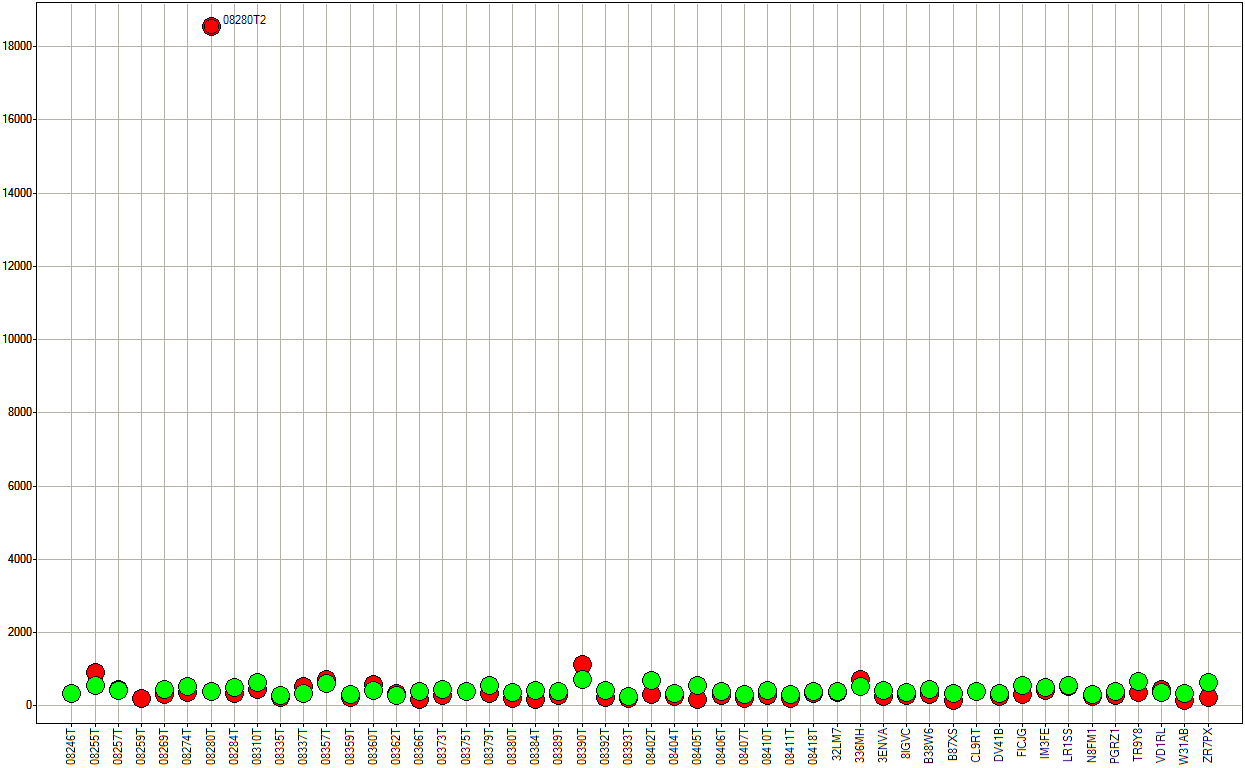
**

**
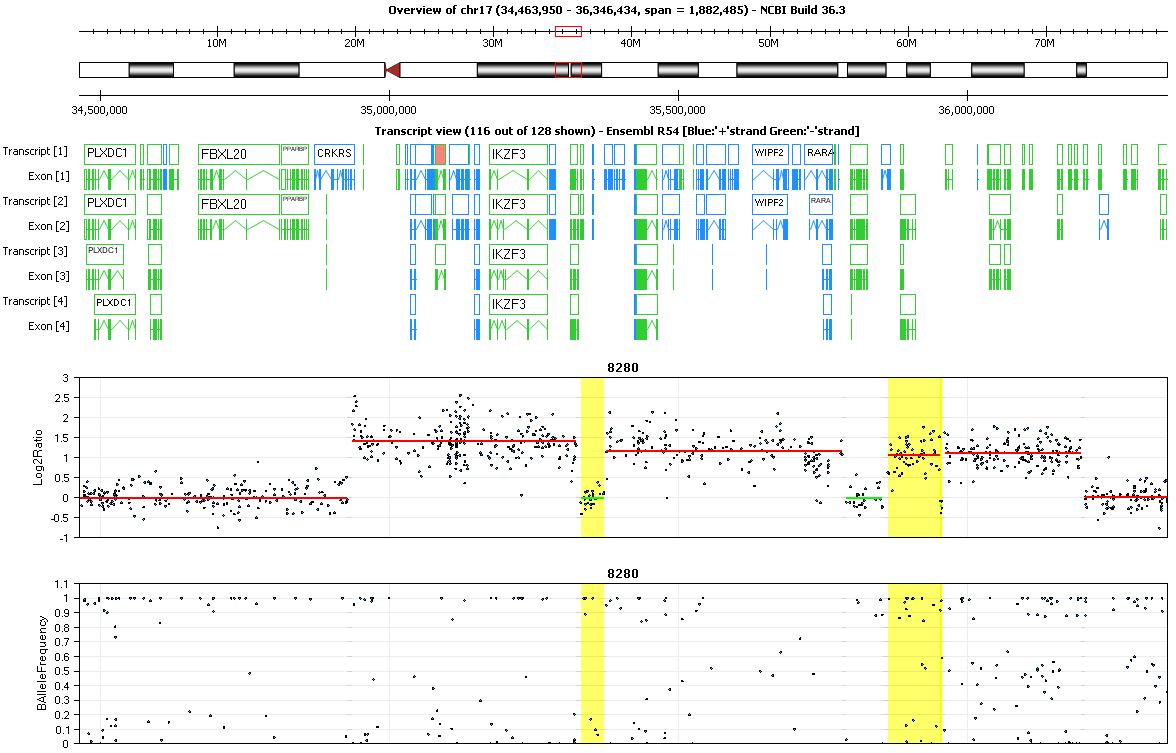
**

Supplement: Additional file 5 — Figure S2: Array data evidencing focal amplifications. Top panels show mRNA expression data from arrays, bottom panels show log2 value for DNA abundance in genomic context as derived from SNP arrays. [file 1479-5876-9-119-S5.DOC]

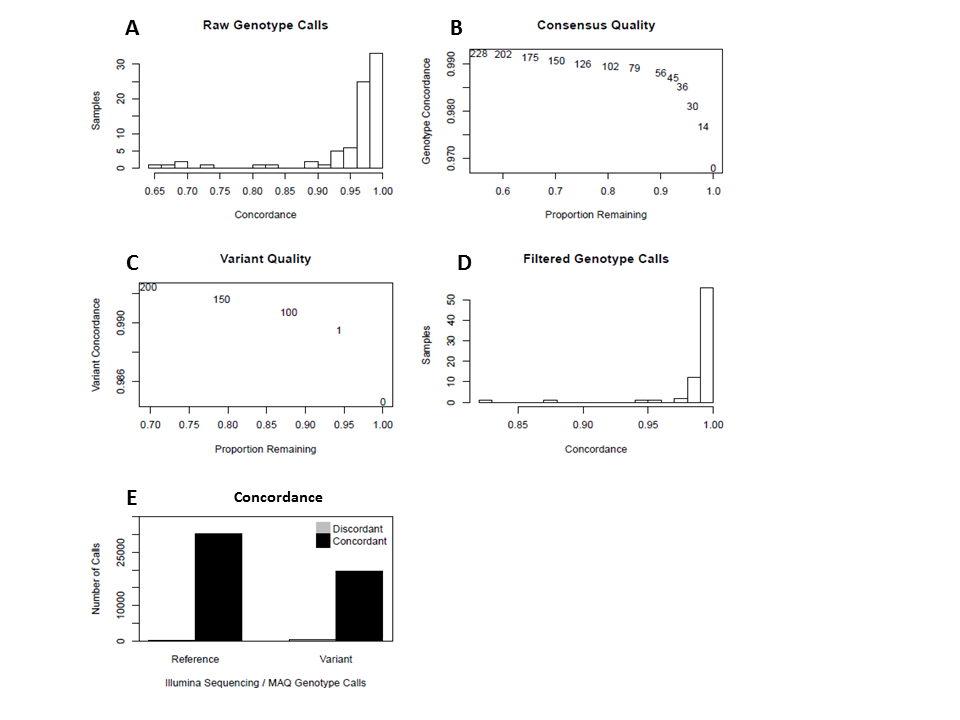

Supplement: Additional file 6 — Figure S3: Comparison of genotyping calls with sequencing data. A total of 1005 common loci were mapped between the Affymetrix 6.0 SNP microarray and the targeted regions. Concordance of genotype calls between affymetrix 6.0 SNP and SAMtools with no filters applied (top left). Application of a consensus quality filters (threshold values plotted as points) improves concordance (y-axis) but reduces the total number of calls (x-axis)(top right). A similar trend is observed for the variant quality thresholds, but at different threshold values (plotted points)(middle left). Sample concordance of genotype calls is improved with consensus quality filter >= 50 and variant quality > 0 (middle right). The total number of genotype calls stratified by reference or variant genotype, and concordance (bottom left). [file 1479-5876-9-119-S6.PNG]

**Figure S2**

**
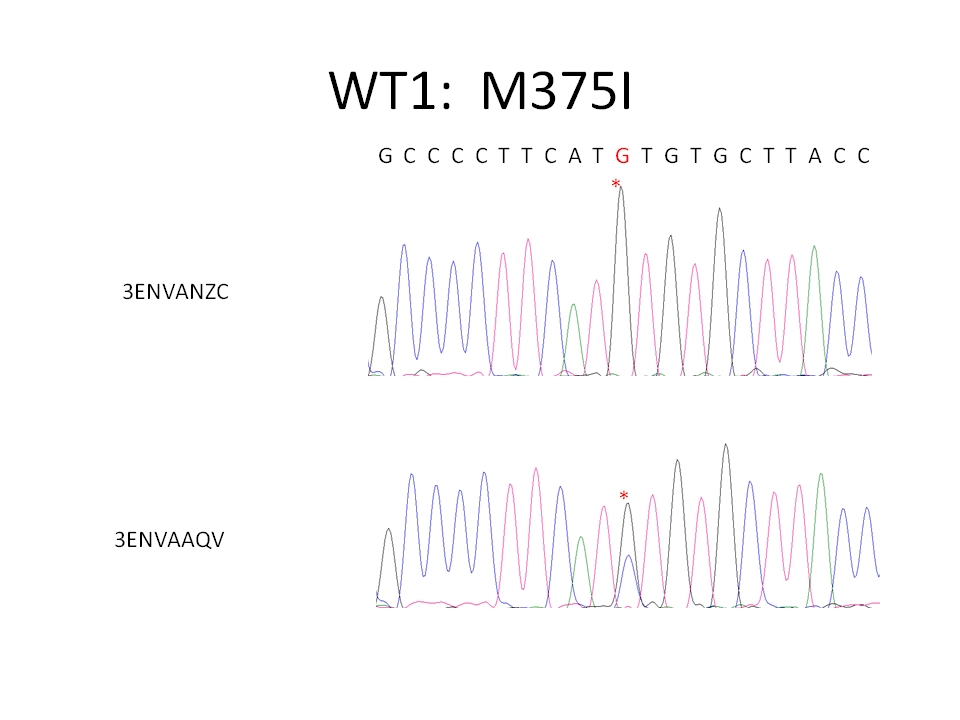

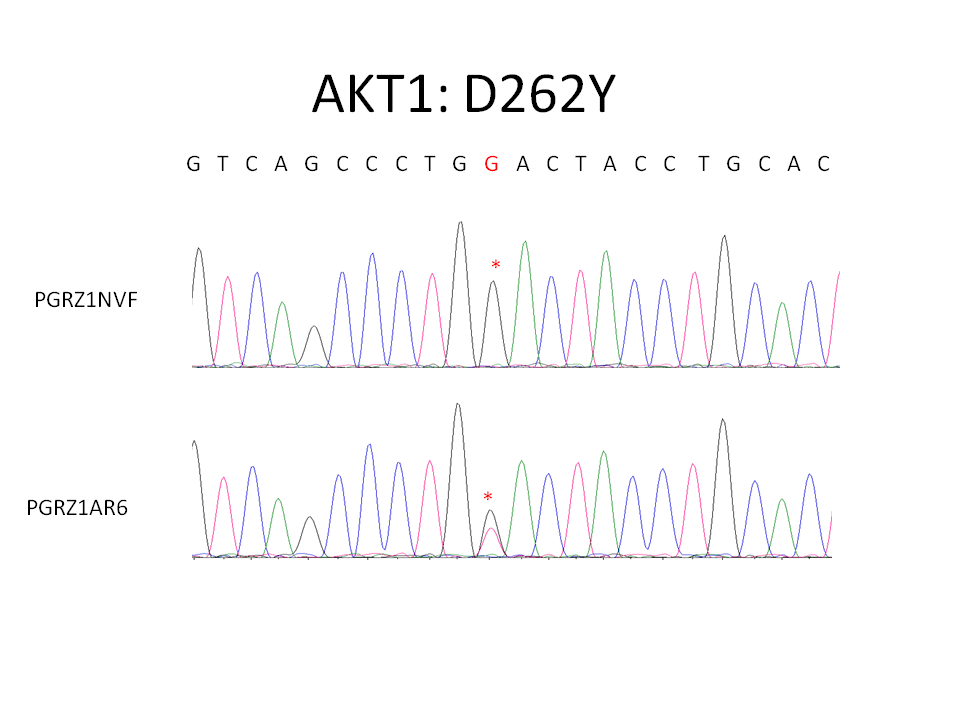

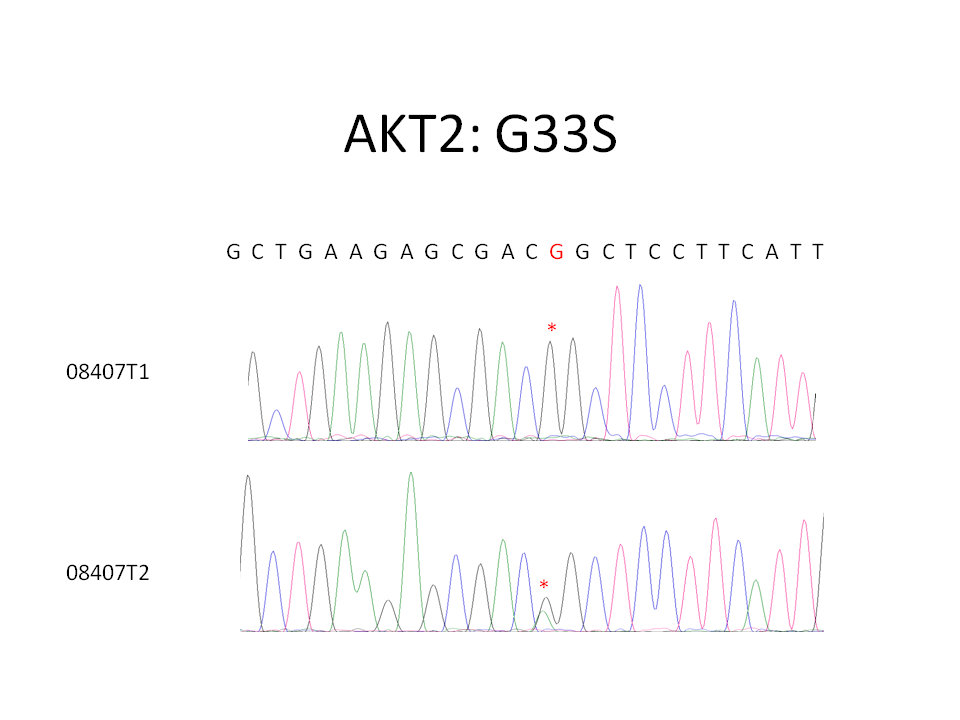

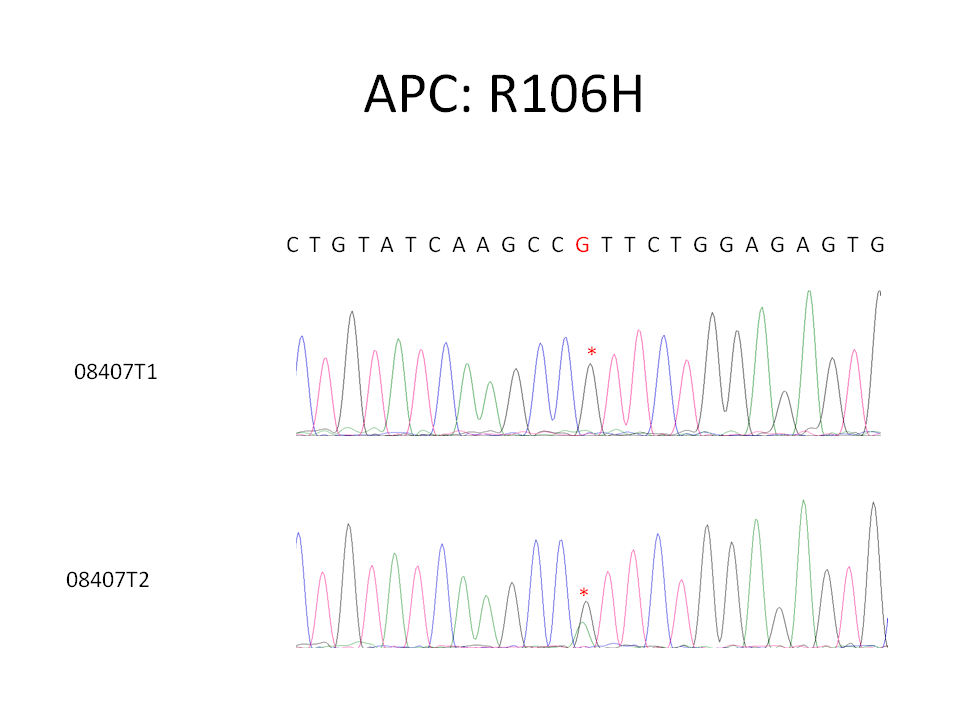

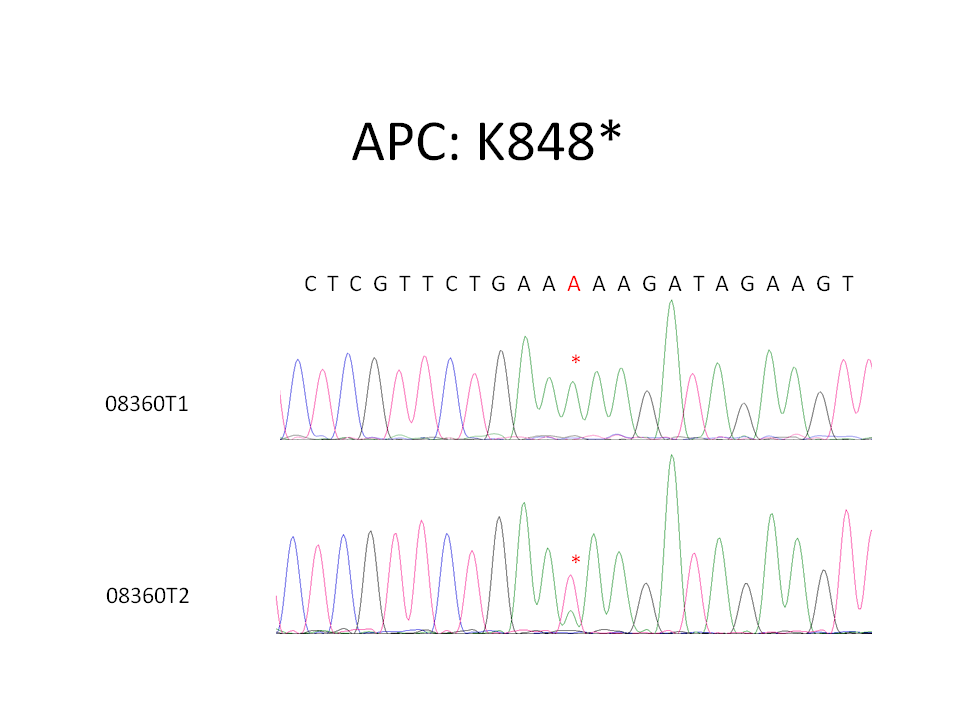

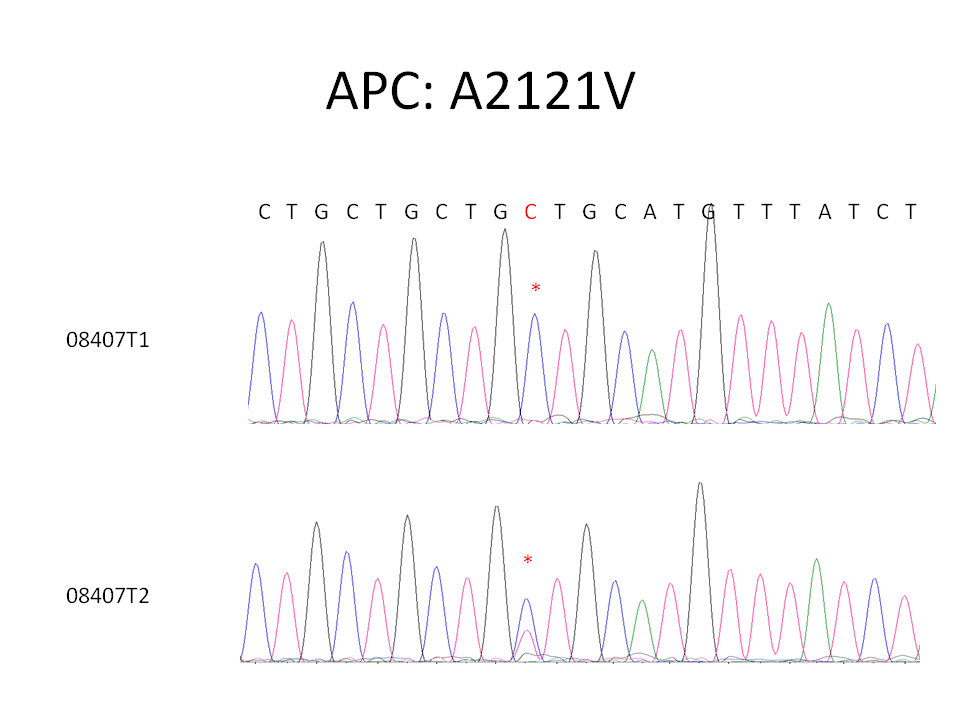

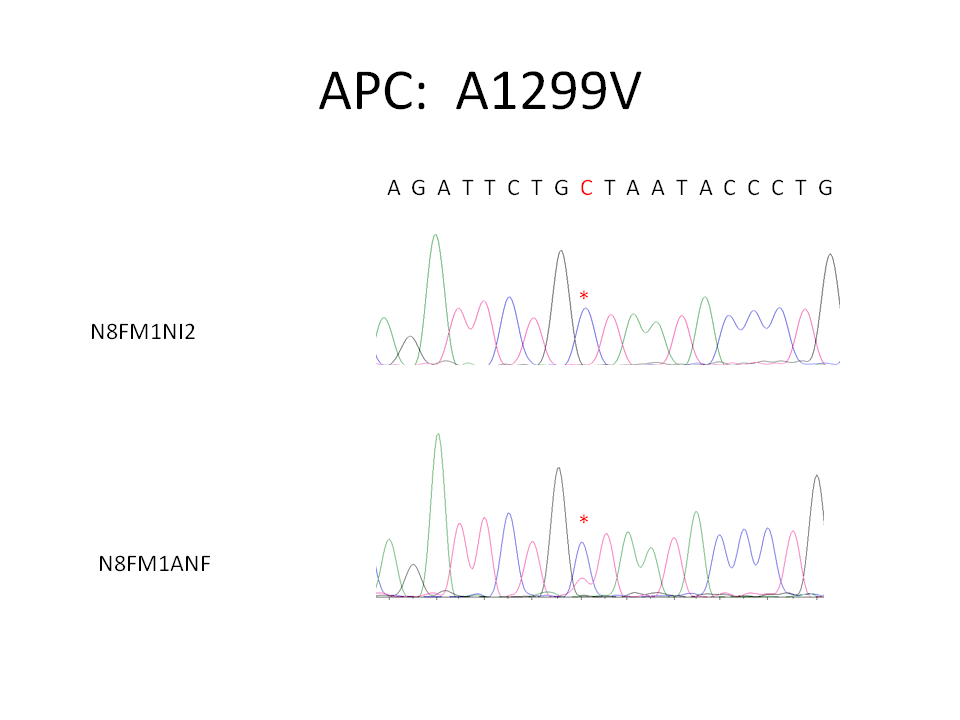

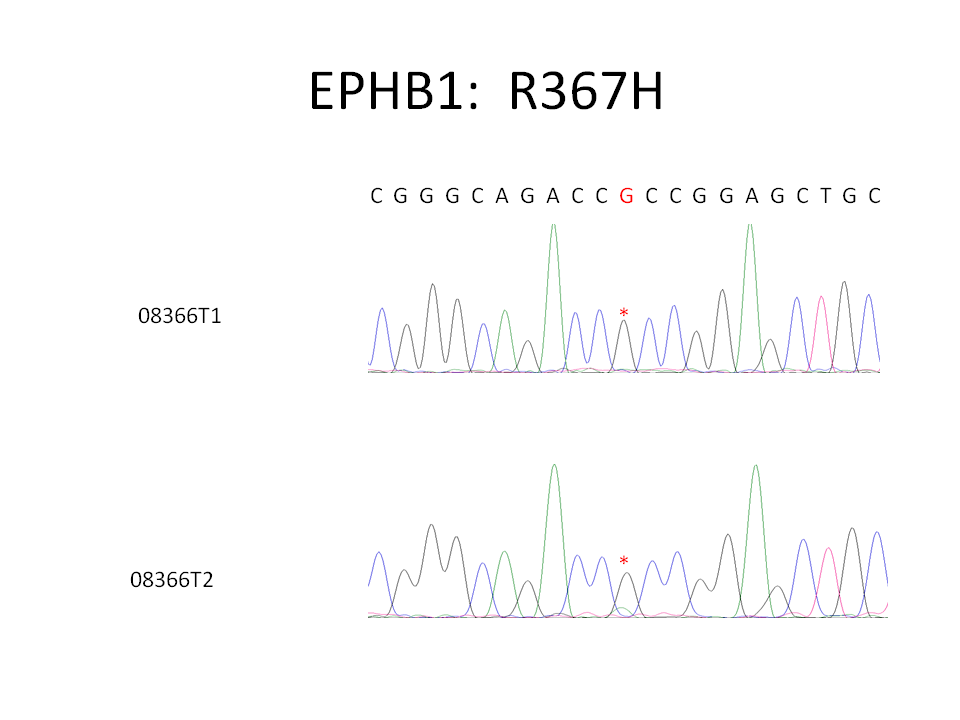

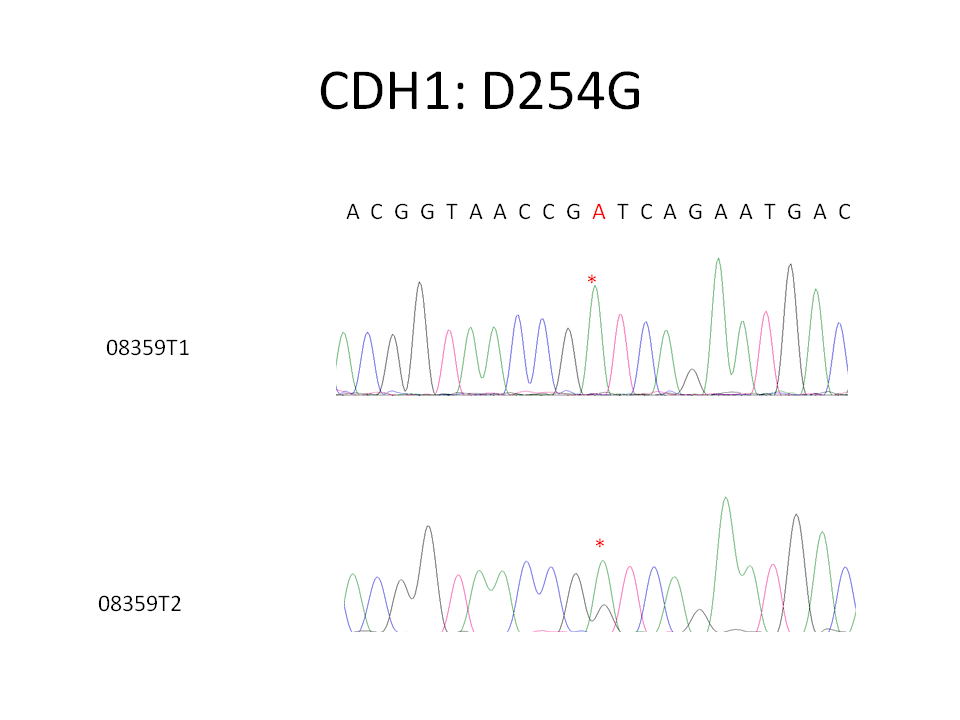

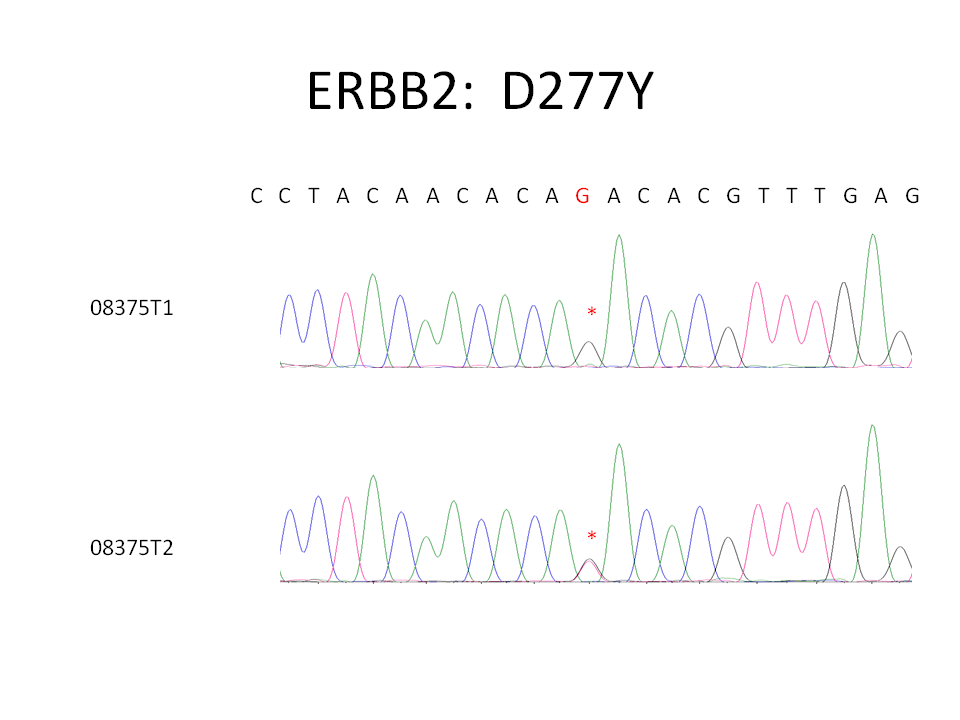

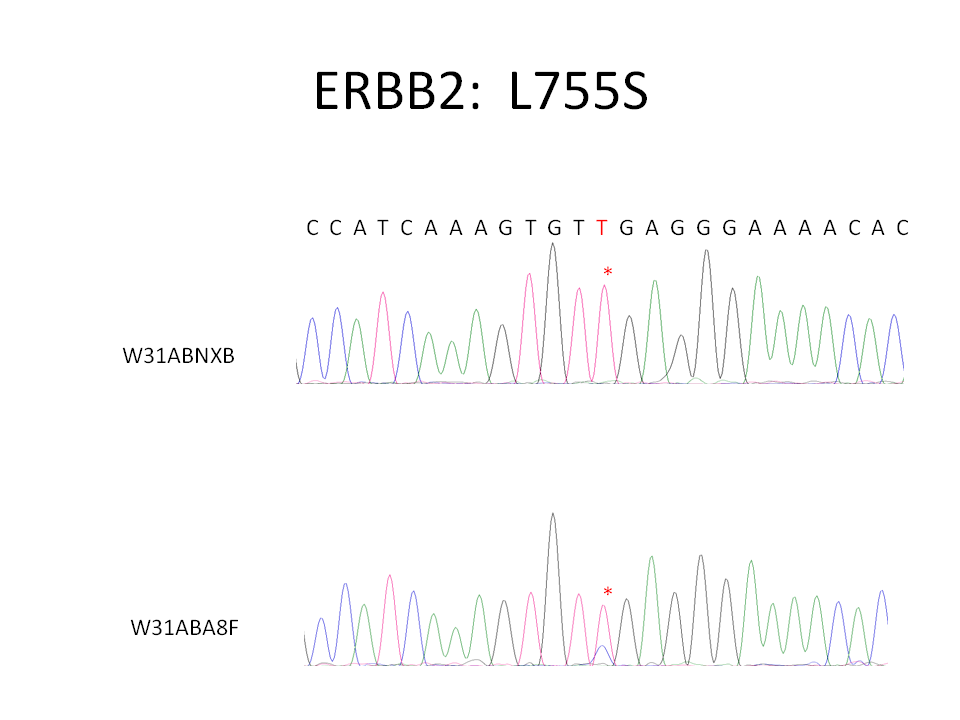

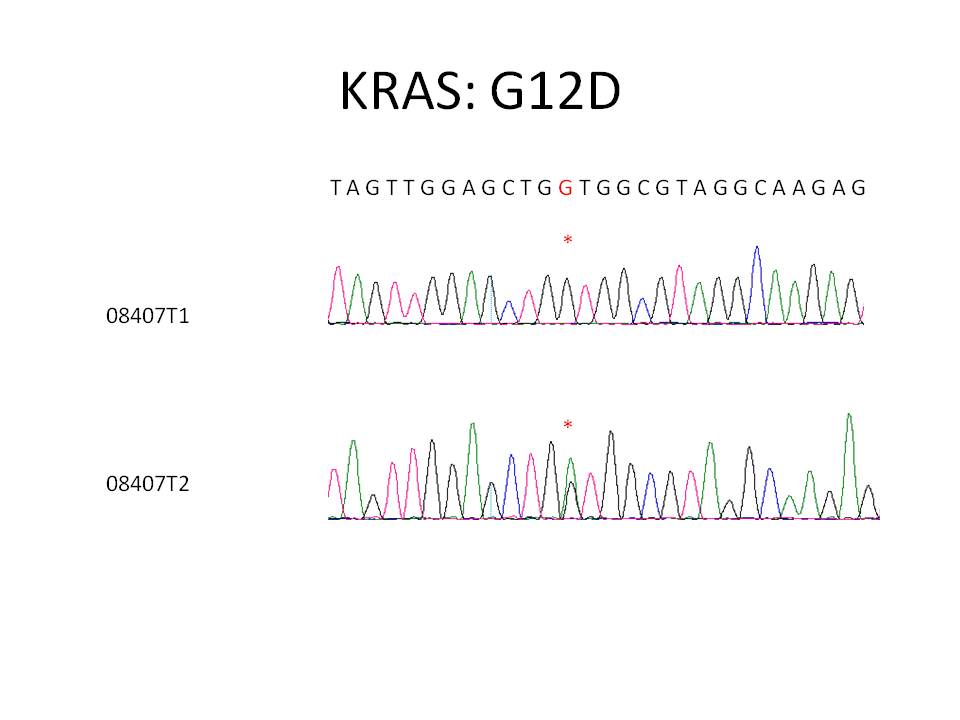

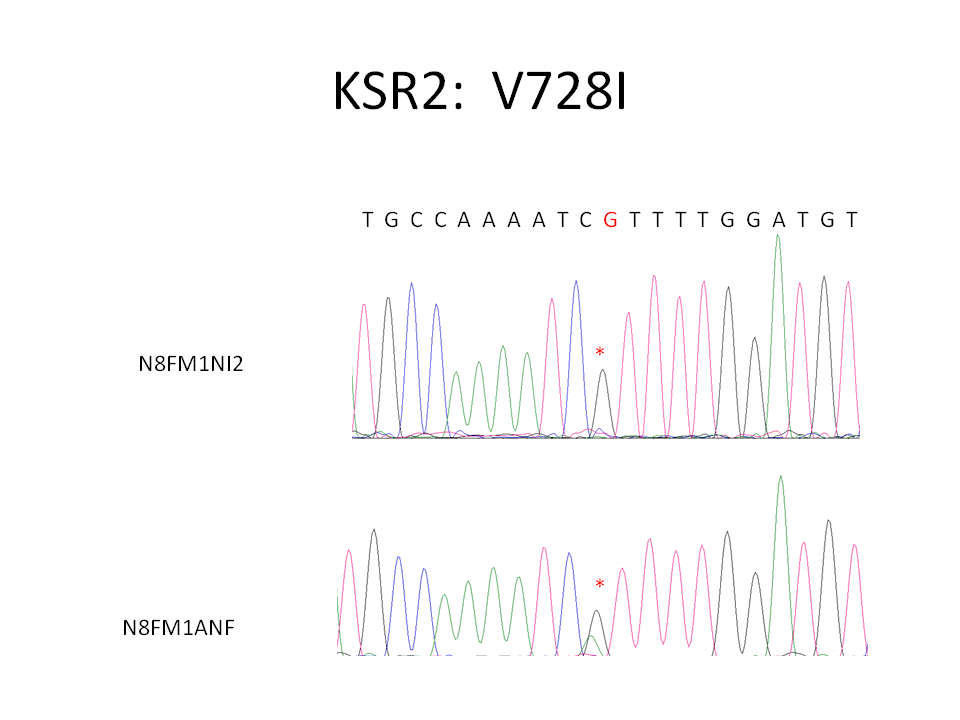

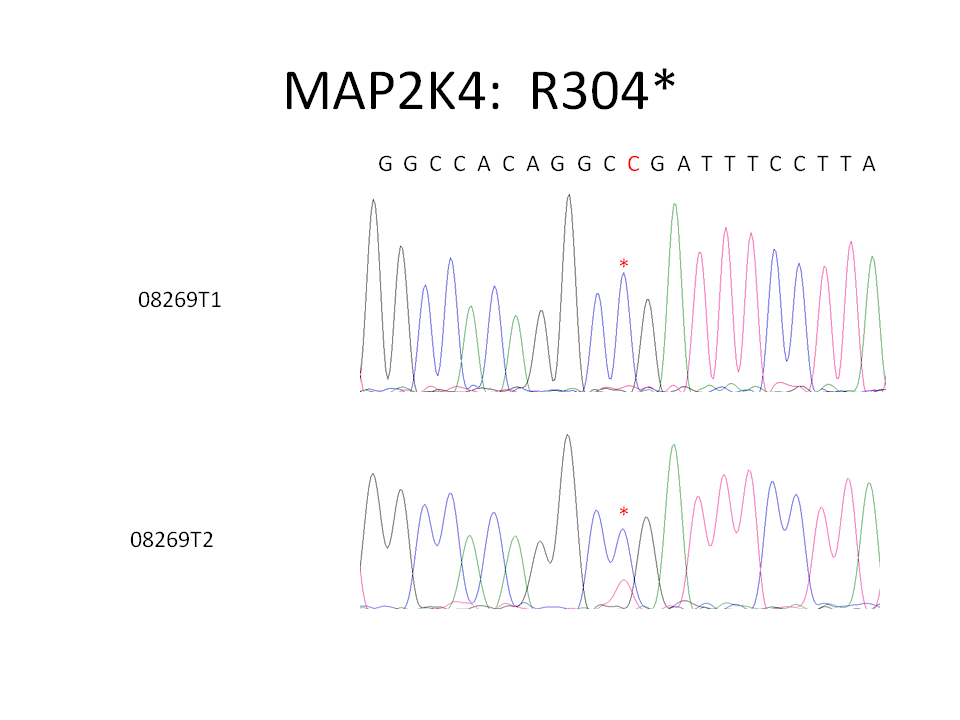

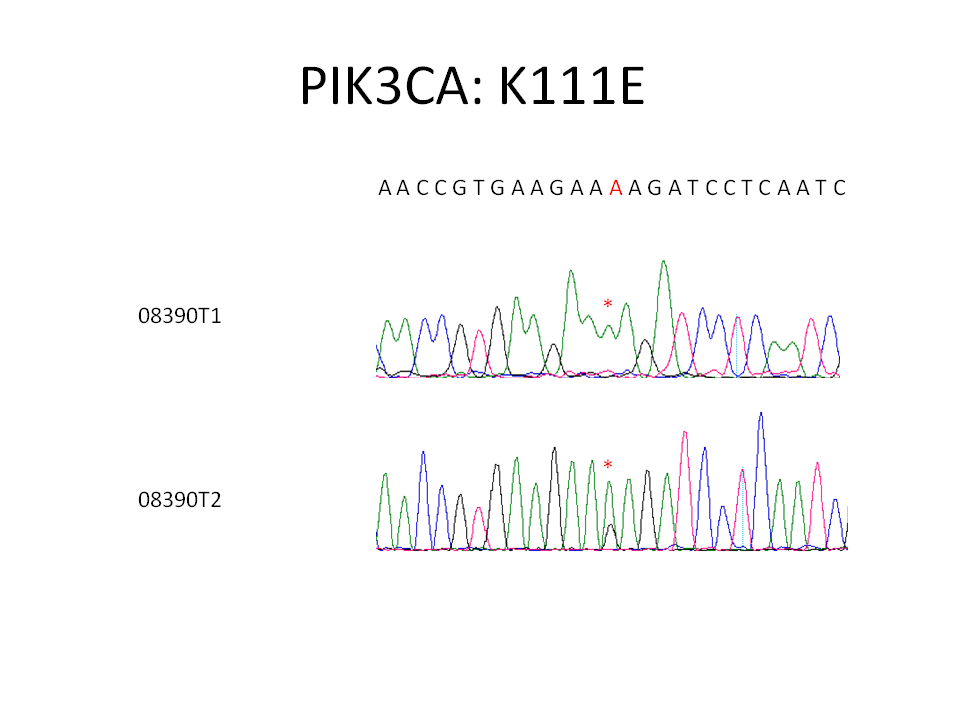

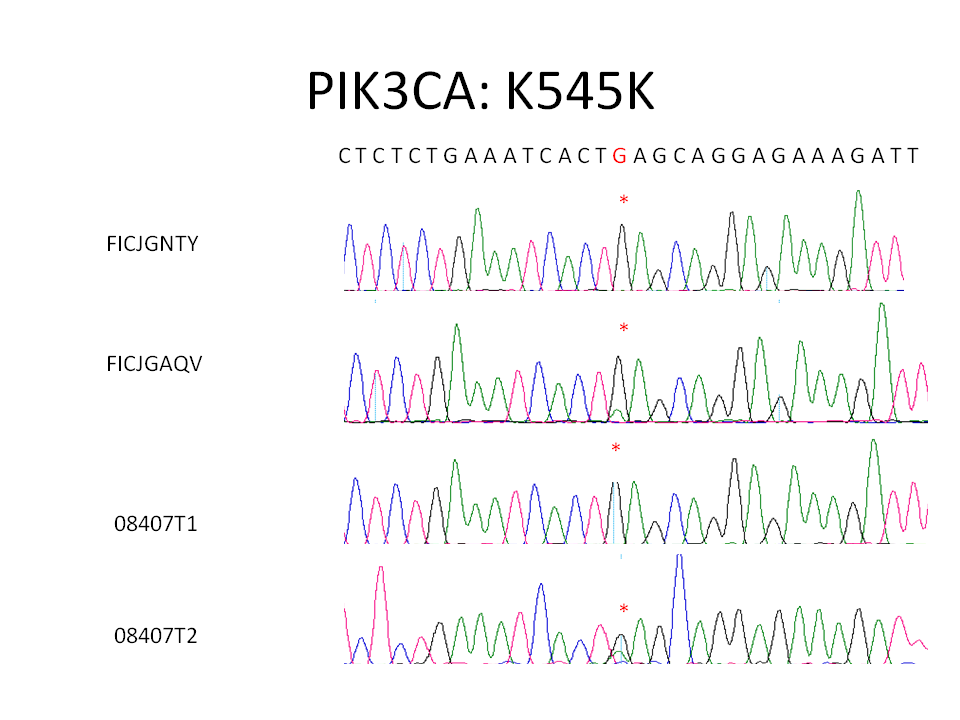

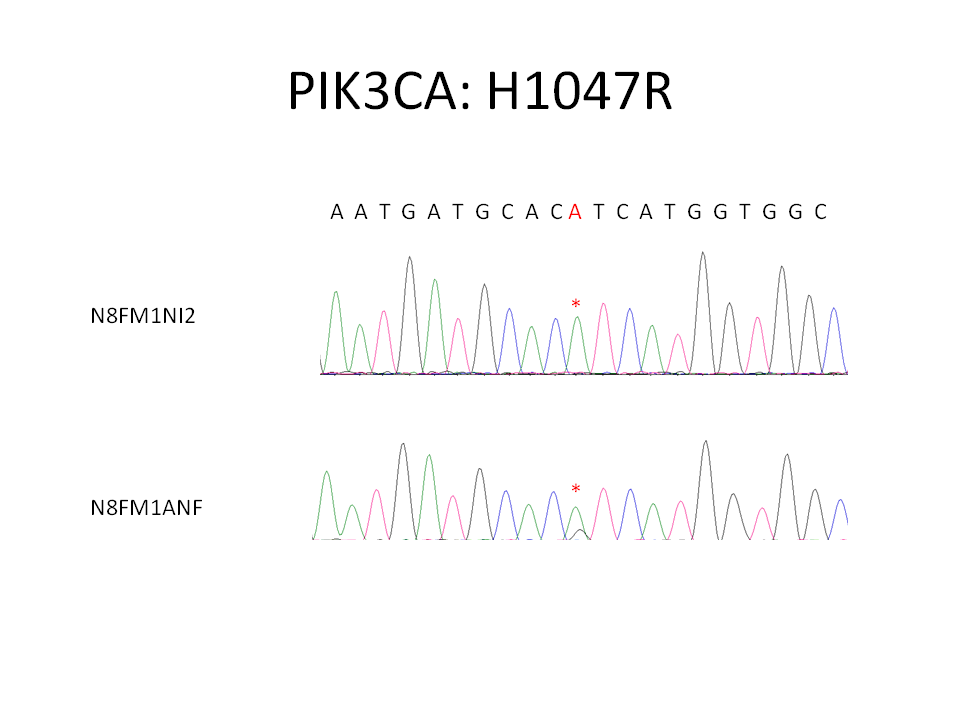

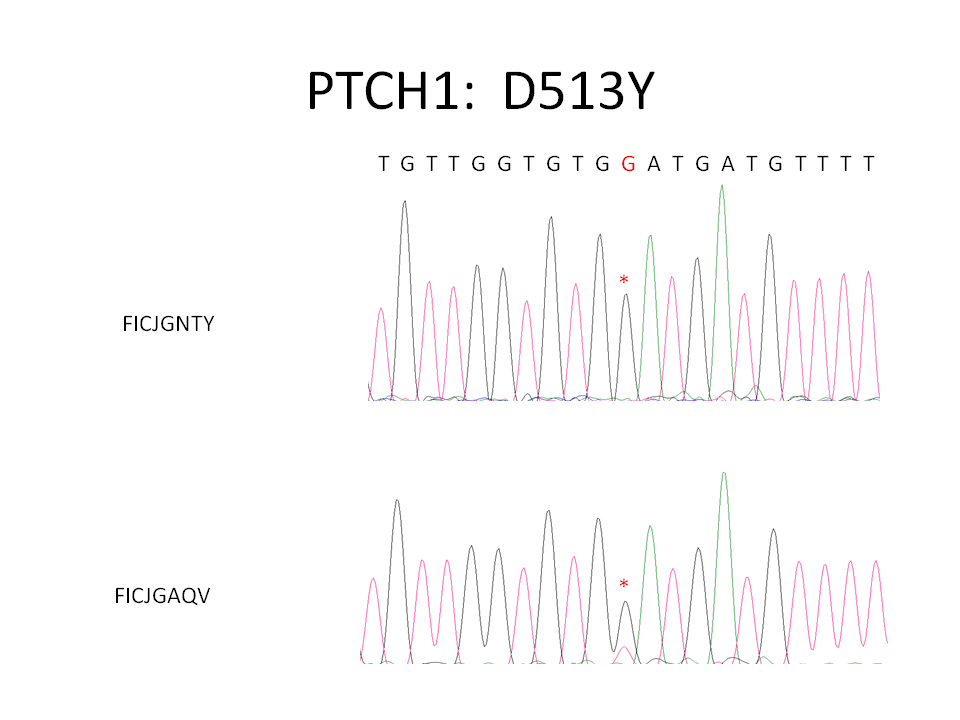

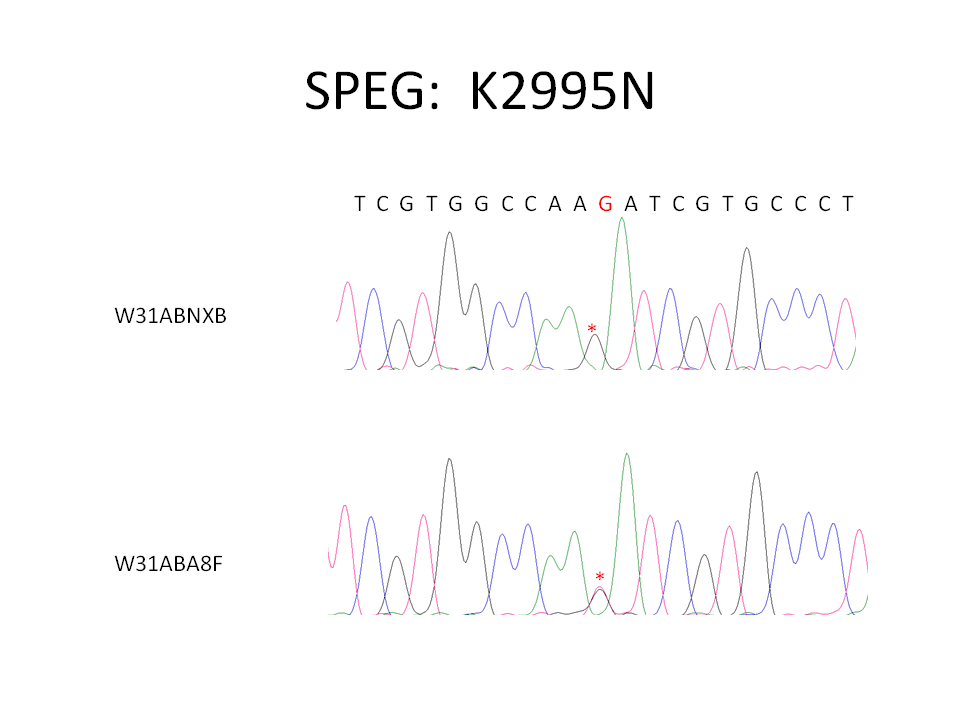

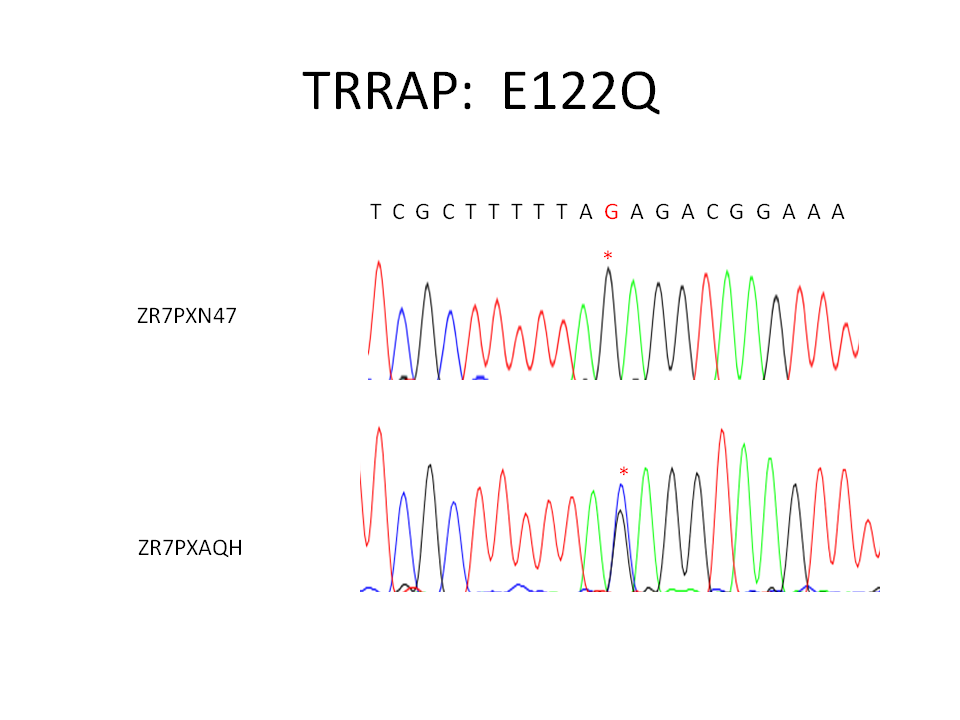

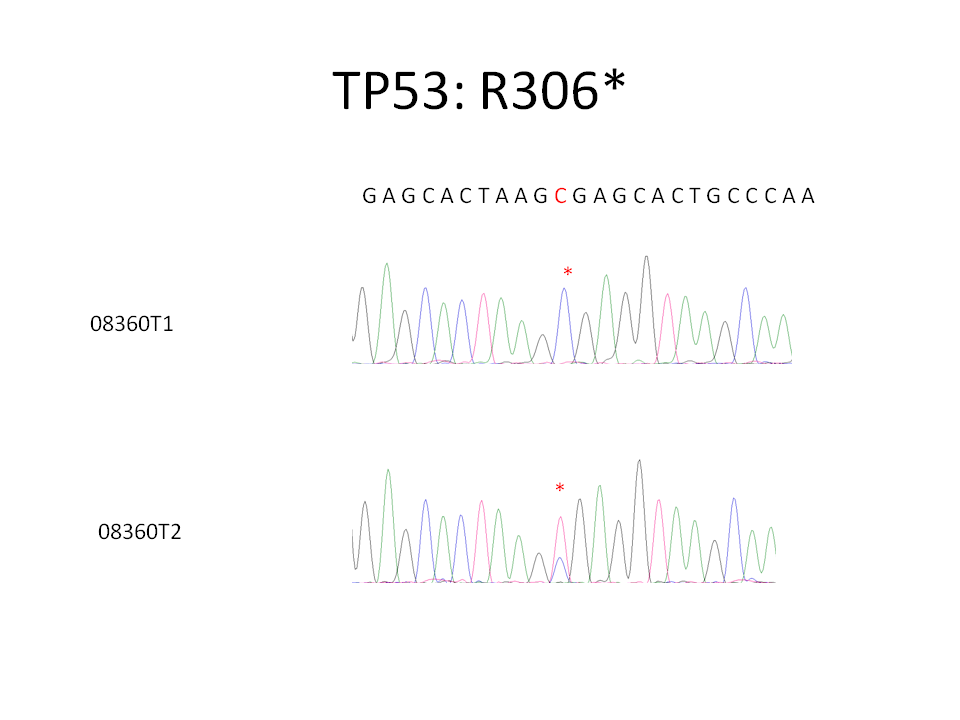

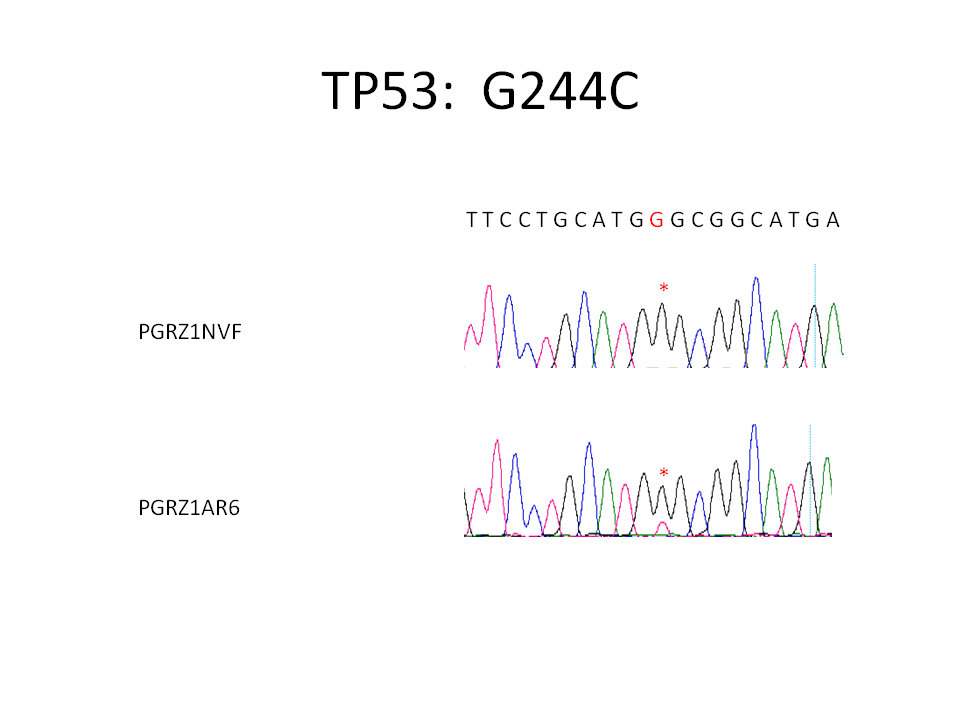

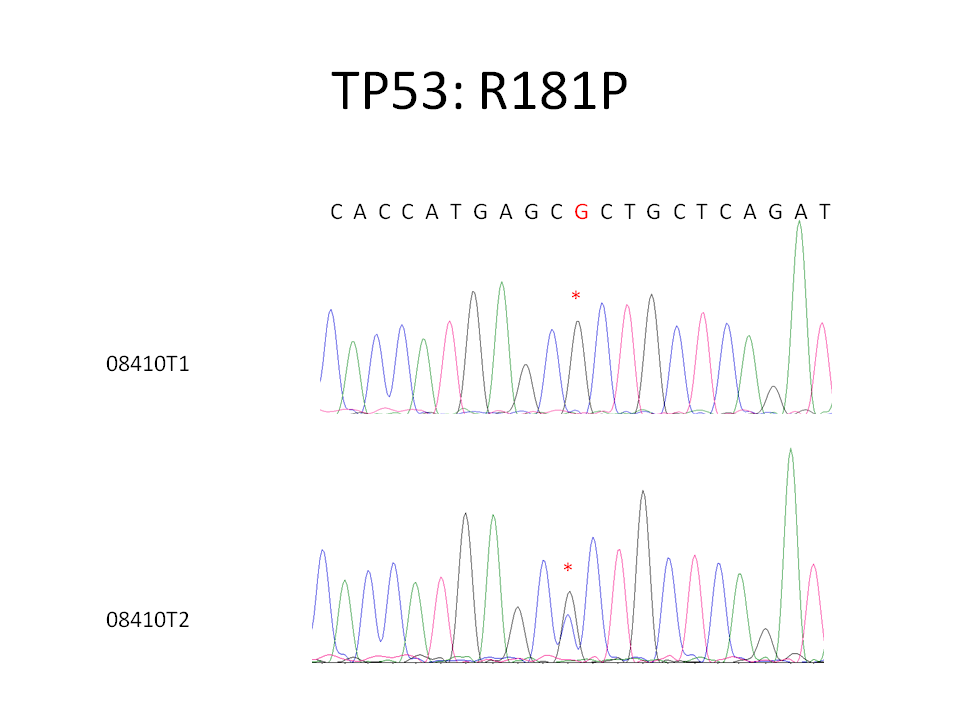

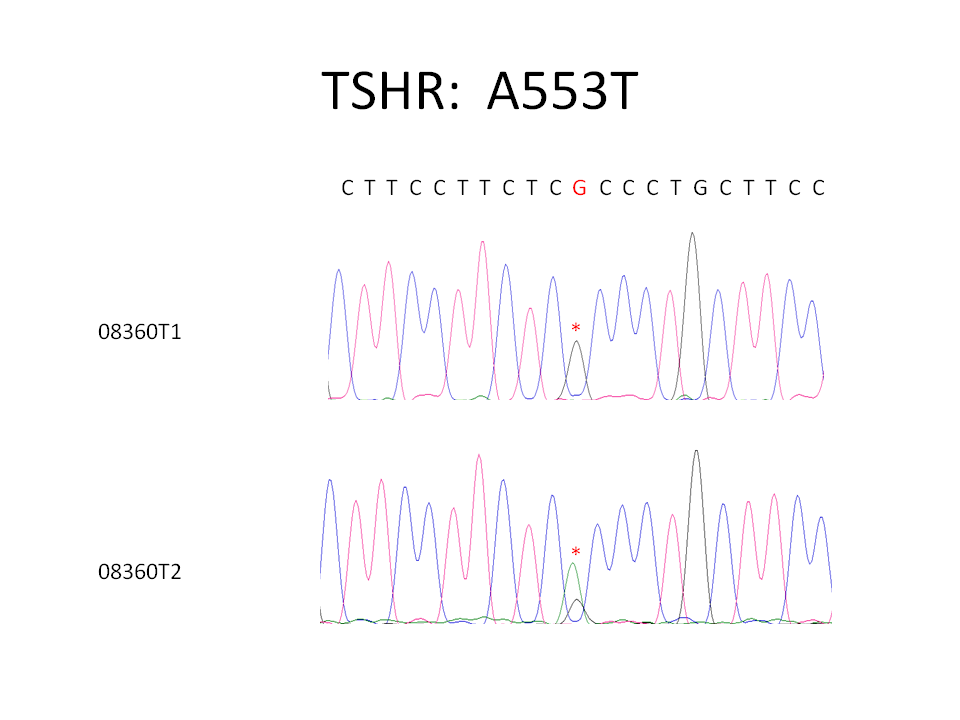

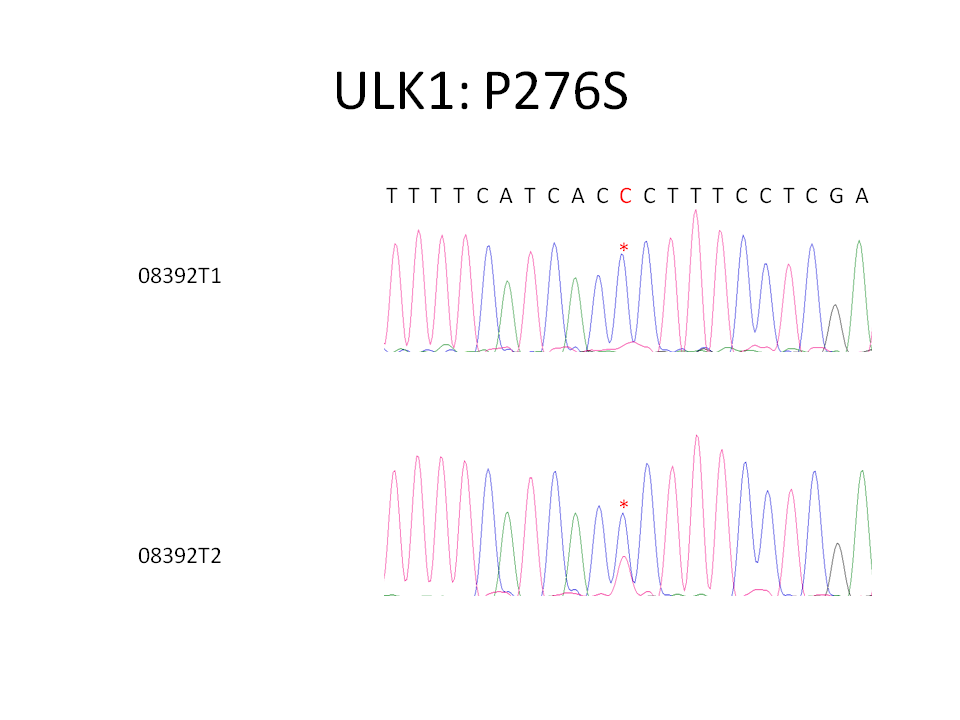
**

Supplement: Additional file 8 — Figure S4: Sanger sequencing traces. Sanger sequencing traces for variants denoted by blue boxes in Figure 3 (i.e. confirmed in Illumnia and Sanger) are provided. [file 1479-5876-9-119-S8.DOC]
